# Lipid flipping in the omega-3 fatty-acid transporter

**DOI:** 10.1038/s41467-023-37702-7

**Published:** 2023-05-08

**Authors:** Chi Nguyen, Hsiang-Ting Lei, Louis Tung Faat Lai, Marc J. Gallenito, Xuelang Mu, Doreen Matthies, Tamir Gonen

**Affiliations:** 1 Howard Hughes Medical Institute, University of California Los Angeles, Los Angeles, CA 90095 USA; 2 Department of Biological Chemistry, University of California Los Angeles, Los Angeles, CA 90095 USA; 3 Janelia Research Campus, Howard Hughes Medical Institute, 19700 Helix Drive, Ashburn, VA 20147 USA; 4 Unit on Structural Biology, Division of Basic and Translational Biophysics, Eunice Kennedy Shriver National Institute of Child Health and Human Development, National Institutes of Health, Bethesda, MD 20892 USA; 5 Molecular Biology Institute, University of California, Los Angeles, Los Angeles, CA 90095 USA; 6 Departments of Physiology, University of California Los Angeles, Los Angeles, CA 90095 USA

**Keywords:** Electron microscopy, Membrane proteins, Membrane structure and assembly, Permeation and transport

## Abstract

Mfsd2a is the transporter for docosahexaenoic acid (DHA), an omega-3 fatty acid, across the blood brain barrier (BBB). Defects in Mfsd2a are linked to ailments from behavioral and motor dysfunctions to microcephaly. Mfsd2a transports long-chain unsaturated fatty-acids, including DHA and α-linolenic acid (ALA), that are attached to the zwitterionic lysophosphatidylcholine (LPC) headgroup. Even with the recently determined structures of Mfsd2a, the molecular details of how this transporter performs the energetically unfavorable task of translocating and flipping lysolipids across the lipid bilayer remains unclear. Here, we report five single-particle cryo-EM structures of *Danio rerio* Mfsd2a (drMfsd2a): in the inward-open conformation in the ligand-free state and displaying lipid-like densities modeled as ALA-LPC at four distinct positions. These Mfsd2a snapshots detail the flipping mechanism for lipid-LPC from outer to inner membrane leaflet and release for membrane integration on the cytoplasmic side. These results also map Mfsd2a mutants that disrupt lipid-LPC transport and are associated with disease.

## Abstract (web summary)

Lipid flipping across membrane leaflets are vital yet enigmatic biological processes. Here, five cryo-EM structures provide snapshots to delineate a mechanism of omega-3 fatty acid flipping across the blood brain barrier.

## Introduction

**Fig. 1:**
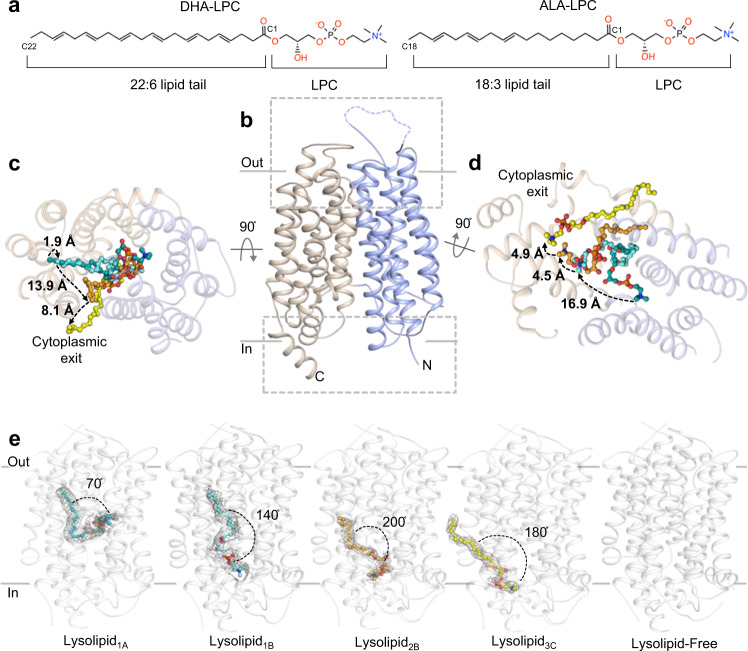
Overall topology and lysolipid transport by drMfsd2a. **a** The preferred Mfsd2a DHA-LPC substrate and the ALA-LPC ligand observed in the drMfsd2a structures. **b** Overall architecture of drMfsd2a with the N-terminal domain, TM1-6, in blue and the C-terminal domain, TM7-12, in wheat. **c** Extracellular view of the substrate translocation pathway with the overlay of the four lysolipid positions observed in drMfsd2a. Dashed lines represent the proposed transport path of the C18 of the lipid tail through a cleft between the N- and C-domains. **d** Cytoplasmic view of the substrate translocation pathway with the overlay of the four lipid positions observed in drMfsd2a through a cleft between the N- and C-domains. Dashed lines represent the proposed transport path of the choline of the LPC headgroup. **b**–**d** Ligands illustrated as stick and sphere. **e** The ligand-free and the four elongated lipid-like densities fitted with ALA-LPC observed for drMfsd2a starting from the position closest to the extracellular towards the cytoplasmic side. Protein rendered as gray cartoon. Elongated densities shown as gray mesh. Lipid isomerism cannot be ascertained at the current resolution.

Docosahexaenoic acid (DHA, 22:6) is an important component of cellular membranes where it forms structural and functional interactions with cholesterol and integral membrane proteins^1^ (Fig. 1a). DHA is highly enriched in the central nervous system (CNS), particularly at neuronal synapses^2^. Inadequate DHA results in several CNS-related ailments including learning and memory deficits, dyspraxia, and dyslexia^3,4^. Despite its essential roles in the CNS, the brain lacks robust mechanisms to biosynthesize DHA, which is estimated to have high turnover rates on the order of minutes^5^. Instead, the brain acquires DHA from plasma by transport across the blood brain barrier (BBB)^6^. Although many studies have gleaned important insights into the roles of tight junctions at the BBB^7–9^, recent work have revealed that DHA is delivered to the brain via selective transport through endothelial cells^5,6^.

Phospholipids can move laterally in membranes but they cannot easily flip vertically between leaflets of the bilayer. Phospholipid flipping between membrane bilayers is energetically costly because this process entails the translocation of a charged headgroup across the hydrophobic region of the bilayer to realign with the opposite leaflet while maintaining the integrity of the membrane. Cells accomplish lipid flipping to establish membrane leaflet asymmetry by specialized transporters termed floppases^10^ and flippases^11–13^. These proteins couple lipid transport and flipping with energetically favorable processes such as ATP hydrolysis or shuttling ions down their electrochemical gradients^11–14^.

The major facilitator superfamily (MFS) is a large class of membrane proteins that span all kingdoms of life having diverse functions and substrate specificities^15,16^. MFS comprise over 25% of all transmembrane proteins and play key roles in cellular physiology. Although members of MFS share low sequence identity, they all share a similar twelve transmembrane helix (TM) topology and use the “rocker-switch” mechanism to transport cargos across membranes^16,17^. The MFS domain containing protein-2a (Mfsd2a) was initially discovered as an orphan receptor in 2008^18^ and later revealed as the primary DHA flippase across the BBB^6^. Mechanistic studies revealed that Mfsd2a is a symporter for Na^+^ and is selective towards long chain, unsaturated fatty acids including DHA (22:6), α-linolenic acids (ALA, 18:3), and oleic acids (18:1) that are covalently tethered to the zwitterionic lysophosphatidylcholine (LPC) headgroup^6,19^ (Fig. 1a). Since most flippases so far are P4-ATP-dependent flippases, Mfsd2a is one of few known flippases outside this family^6,11^. The three other known MFS lipid transporters are Mfsd2b^20^, LplT^21^, and Spns2^22^; however, they confer different substrate specificities and their transport mechanisms are yet to be defined. Importantly, Mfsd2a is required for the formation, development, and function of both the BBB and the CNS^23–25^. Defects in Mfsd2a disrupt normal brain development and function resulting in phenotypes including anxiety, learning deficits, ataxia, and severe microcephaly^6,23,25–27^. Consistent with these findings, it was recently demonstrated that Zika virus causes microcephaly by proteolytic degradation of Mfsd2a^28^.

Even with recently published structures of Mfsd2a^19,29,30^, the molecular details of how Mfsd2a performs lipid-LPC flipping and transport across the lipid bilayer remains obscure. Here, we present five structures of the Zebrafish (*Danio rerio*) isoform B Mfsd2a (drMfsd2a) in the inward-facing conformation determined by single-particle cryo-electron microscopy (cryo-EM), in the ligand-free state and displaying lipid-like densities at four distinct positions modeled as ALA-LPC (Fig. 1b–e). These four modeled ligand configurations allowed elucidation of (1) a clearer step-by-step transport and flipping mechanism of lipid-LPC, and (2) the release of the substrate to the cytoplasmic side of drMfsd2a for inner membrane leaflet integration (Fig. 1c–e). The details gleaned from these studies provide the molecular models of the proposed drMfsd2a lipid, phosphate, and choline binding sites, and how these components are orchestrated during the transport and flipping of the lysolipid. We also identify the interactions between the modeled lipid-LPC and drMfsd2a at four distinct positions, and map key residues that when mutated (1) disrupt lysolipid transport, and (2) are associated with known Mfsd2a-related diseases. Together this study adds to the repertoire of transport mechanisms by MFS by generating models for how members of this superfamily of proteins transport lipid cargos. Given its roles in brain function and development, our study lays the foundation for better understanding of neurological conditions and motor dysfunctions linked to defects in this family of transporters.

## Results

### Overall topology of drMfsd2a in complex with lipids

DrMfsd2a is a small protein at 59 kDa and structures of this size are exceedingly challenging to determine by single-particle cryo-EM^31^. As such, we generated and formed a complex between a FAB^32^ and drMfsd2a to facilitate cryo-EM structure determination (Supplementary Figs. 1–5, Supplementary Table 1). Further, alignment between the human and drMfsd2a sequences revealed a 76% similarity and 61% identity (Supplementary Fig. 6). Given that most MFS members share low sequence identities but a conserved fold^15^, sequence conservation between these two homologs suggests that the zebrafish protein can serve as a model for understanding human Mfsd2a.

Like other MFS members, drMfsd2a is comprised of twelve TMs that pack against one another with the substrate binding channel located at a cleft between the N- and C-terminal domains (Fig. 1b). The twelve TMs of drMfsd2a are, likewise, subdivided into two helical bundles called the N- (helices 1–6) and C-domains (helices 7–12) that are related by a twofold pseudosymmetry (Fig. 1b, Supplementary Fig. 6). This topology is consistent with other MFS members that have been structurally described, including the recent chicken^19^, mouse^29^, and human^30^ Mfsd2a structures. These three Mfsd2a structures were solved in the outward-facing, ligand-free conformation from mouse^29^, outward-occluded ligand-free state in complex with the placenta SYNC2 protein from human^30^, and the inward-facing complex bound with ALA-LPC from chicken^19^. These three structures are distinct from   this study because none delineated the lipid flipping function of the transporter (Fig. 1d, e, Supplementary Fig. 7). Here, we report five structures of drMfsd2a in the inward-facing conformation in the ligand-free state and    with bound lipid at four distinct locations in the substrate translocation tunnel (Fig. 1). We expressed and purified drMfsd2a from SF-9 insect cells and discovered that the protein copurified with endogenous phospholipids (Supplementary Fig. 8a–c). These results are consistent with past reports of Mfsd2a copurifying with native ALA-LPC from SF-9 insect cells^19^. Functional assays using reconstituted proteoliposome coupled with [^14^C]DHA-LPC uptake further confirmed that drMfsd2a is indeed active and able to bind lipids  (Supplementary Fig. 9). We therefore modeled ALA-LPC into the four distinct lipid-like densities termed Lysolipid_1A, 1B, 2B, 3C_ (numbers and letters refer to the lipid and LPC binding positions) (Fig. 1e, Supplementary Figs. 5, 10, 11). 

Initial data processing yielded a 2.9 Å resolution map with three lipid-like densities representing three merged ligands, Lysolipid_1A, 2B, 3C_ (Fig. 1e). However, our in vitro analysis indicates that there is only ~1 phospholipid per protein molecule (Supplementary Fig. 8c), suggesting that the lipids observed in this model is a merge of particles of substrates bound at three separate sites. Due to the small size of the phospholipid, traditional classification and 3D variability analysis^30^ were unable to separate individual states. Alternatively, we deployed 3D classification without alignment in cryoSPARC with five input references including Mfsd2a-FAB with (1) Lysolipid_1A, 2B, 3C_, (2) no ligand, (3) Lysolipid_1A_, (4) Lysolipid_2B_, and (5) Lysolipid_3C_ bound (Supplementary Fig. 2). Maps with individual lipid-like densities were obtained after classification (Supplementary Figs. 2, 4, 5). To mitigate reference bias, reconstruction of each subclass was performed through ab-initio model building and non-uniform refinement with initial model lowpass-filtered to 30 Å, eventually yielding maps at resolutions from 3.3 to 3.4 Å (see methods). To gain more accurate assessment of how drMfsd2a coordinates these ligands, we plotted the intermolecular Lysolipid_2C_- and Lysolipid_3C_-drMfsd2a interactions using the model for the merged ligand map at 2.9 Å resolution (Table S2). A reference with a segment of TM1 truncated was included as a control (Supplementary Fig. 3). Recovery of TM1 segment density from classification and refinement indicates validity of the image processing scheme. We also obtained the lipid-like density for Lysolipid_1B_ using an alternate strategy for particle picking, 2D classification, and 3D reconstruction (see methods).

**Fig. 2:**
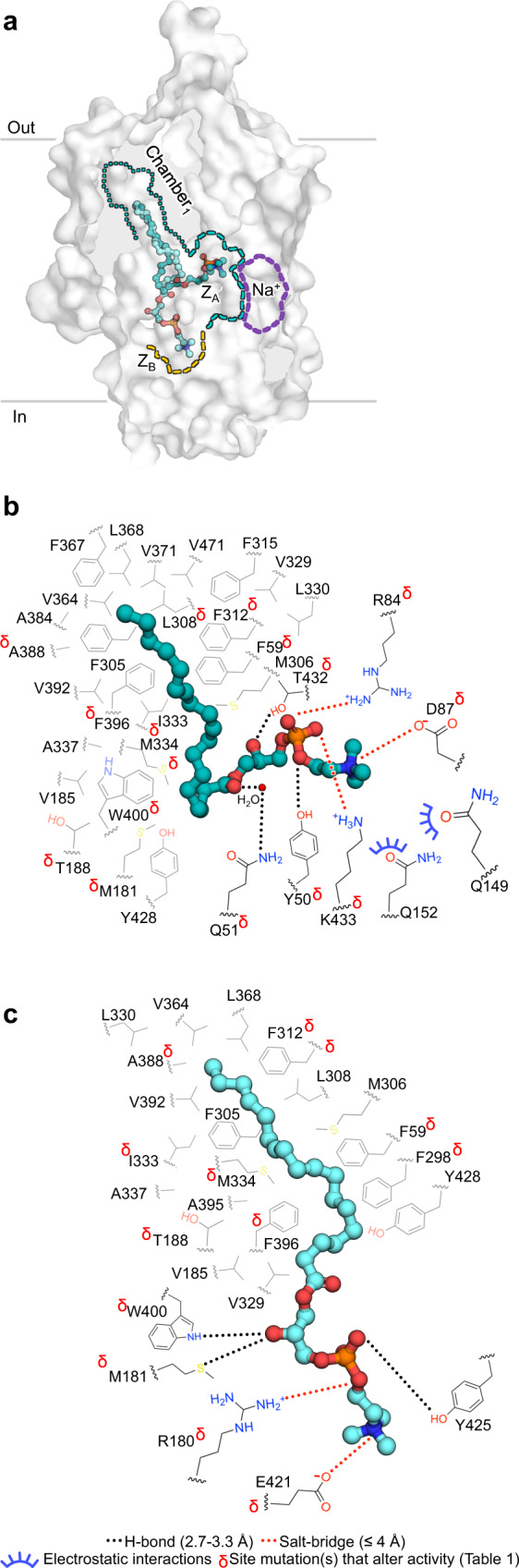
Rotation of the LPC headgroup. **a** Relative positions of Lysolipid_1A-1B_ in Chamber_1_ and Z_A-B_. Lipid Chamber_1_ is outlined in cyan dotted lines. Z_A-B_ are in dashed cyan and orange lines, respectively. The proposed Na^+^ binding site is highlighted by dashed purple lines. Protein rendered as surface. Lysolipids_1A-1B_ are in stick and sphere. **b** Interactions between lipid tail and LPC of Lysolipid_1A_ with residues in Chamber_1_ and Z_A_. **c** Interactions between lipid tail and LPC of Lysolipid_1B_ with residues in Chamber_1_ and Z_B_. **b**, **c** Carbons of lipid chamber residues are in gray. Carbons of Z-site residues are in black. Black dotted lines represent H-bonding between 2.6 Å and 3.3 Å. Red dotted lines indicate salt-bridges with distances ≤4 Å. Blue half circles indicate choline coordinating residues within 3.5 Å. Red δ indicate residues with mutations that alter activity (Table 1). Water molecule shown as small red sphere.

**Fig. 3:**
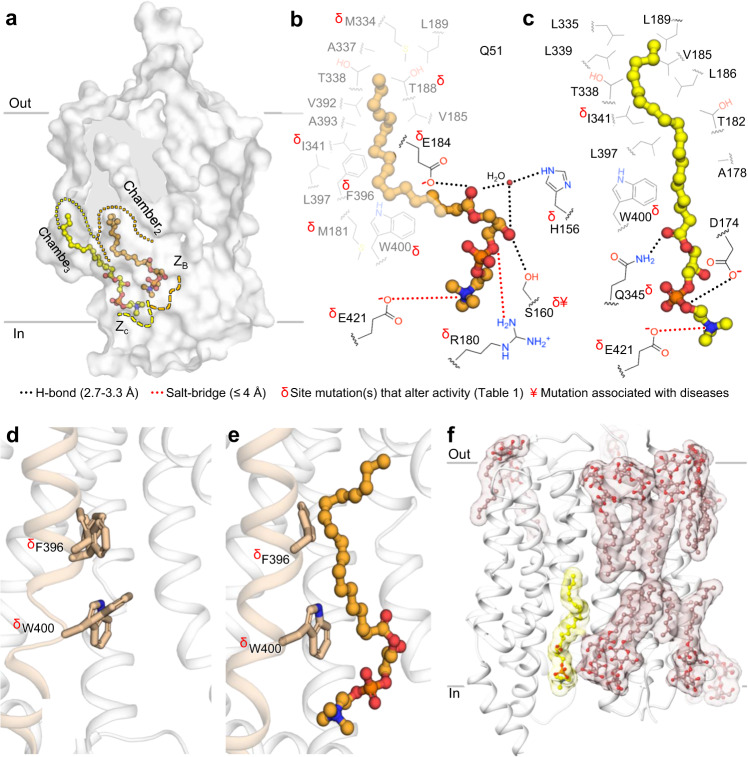
Lysolipid translocation and release through Chamber_2-3_ and Z_B-C_. **a** Relative positions of Lysolipid_2B-3C_ in Chamber_2-3_ and Z_B-C_. Lipid Chambers_2-3_ are outlined in orange and yellow dotted lines, respectively. Z_B-C_ are in dashed orange and yellow lines, respectively. Protein is rendered as surface. Lysolipids are in stick and sphere. **b** Interactions between lipid tail and LPC of Lysolipid_2B_ with residues in Chamber_2_ and Z_B_. **c** Interactions between lipid tail and LPC of Lysolipid_3C_ with residues in Chamber_3_ and Z_c_. **b**, **c** Lipid chamber residues are in gray. Z-site residues are in black. Black dotted lines represent H-bonding between 2.6 Å and 3.3 Å. Red dotted lines indicate salt-bridges with distances ≤4 Å. Red δ and ¥ indicate residues with mutations that alter activity and are associated with disease (see Table 1). Water molecule shown as small red sphere. **d** Substrate transport tunnel in closed conformation. The Lysolipid_1A_ structure is shown with alternate conformations of the F396 and W400 from structures without lipid tail of ALA-LPC in Chamber_2_. **e** Substrate transport tunnel in open conformation with Lysolipid_2B_ bound. **d**, **e** Protein rendered as cartoon. Gating residues are in stick. TM10 colored in wheat. **f** Lysolipid_3C_ (yellow) at the cytoplasmic exit shown adjacent to the DDM belt with detergent molecules shown in stick and ball, and in tan transparent surface.

The maps for drMfsd2a display four densities that cannot be attributed to the proteins chain (Fig. 1e, Supplementary Fig. 5). Closer examination revealed that the elongated densities are surrounded by hydrophobic residues on one side and charged residues at the other end (Figs. 2–3). As such, these elongated densities can each accommodate substrate with a lipid tail and a hydrophilic headgroup (Figs. 2–3, Supplementary Fig. 5). We considered several other ligands that could account for these densities including ALA-LPC, which is known to copurify with Mfsd2a when expressed and purified from SF-9 cells^19^, and DDM and DM which were used during protein purification. ALA-LPC, rather than DM and DDM, provide better fitting into the elongated  densities (Supplementary Fig. 5) and resulted in lower B-factor and more stable binding energy calculations (Supplementary Fig. 10). For Lysolipid_1A_, the density for the headgroup is weaker than that observed for the other ALA-LPC sites (Supplementary Fig. 5a). This is likely because the LPC headgroup of Lysolipid_1A_ is structurally dynamic in an open cavity between the ZA and ZB sites (Fig. 2a). Further, modeling detergent into the elongated density places the hydrophilic sugar rings of the detergent in the unfavorable hydrophobic cavity of Chamber_1_ (Supplementary Fig. 11a). This hydrophobic cavity was also observed for binding of the lipid tail of ALA-LPC in the chicken Mfsd2a structure^20^ and thus unlikely to bind the hydrophilic head of a detergent. It is therefore more likely  that a lipid occupies this site (Supplementary Fig. 11b).

These results are consistent with the observation that ALA-LPC copurifies with the Mfsd2a when purified from SF-9 insect cells^19^. The placement of the four ALA-LPC in the elongated densities in drMfsd2a is unique from where this lysolipid was modelled  in the structure of the chicken Mfsd2a^19^ (Fig. 1, Supplementary Fig. 7c). In drMfsd2a, the four lysolipids are positioned along the vertical axis of the substrate tunnel within a cleft between the N- and C-domains comprise of helices 1, 2, 4, 5, 7, 8, 9, 10, and 11 that open to the cytoplasmic side (Fig. 1b–e, Supplementary Fig. 6). Together, the four modeled ALA-LPC binding positions provide a unique opportunity to detail the transport and flippase mechanism of Mfsd2a as the substrate is shuttled through the transporter. Since Mfsd2a transports lipid-LPC from the outer to the inner leaflet, we propose the following order for the four ALA-LPC during substrate transport through Mfsd2a: Lysolipid_1A,_ Lysolipid_1B_, Lysolipid_2B,_ then Lysolipid_3C_ (Fig. 1e).

### Rocker-switch motion

To gain further insights into the differences between known major conformations of the rocker-switch, we mapped the interdomain interactions observed in the outward-facing mouse^29^, outward-occluded human^30^, and our inward-drMfsd2a structures (Supplementary Movie 1–2). The interdomain interactions in the mouse and drMfsd2a structures include hydrophobic, salt-bridge, and hydrogen bond contacts located on the (1) extracellular end, (2) cytoplasmic side, (3) between helices 5 and 8 and (4) across helices 2 and 11 of Mfsd2a (Supplementary Movie 1). Strikingly, the rocker-switch motion “rocks” along the vertical axes of TM5 and TM8, and TM2 and TM11 (Supplementary Movie 1–2). We also observe that the outward-facing mouse conformation^29^ is held in place by more hydrogen bonds and salt-bridges placed at the opposite, cytoplasmic side than observed for interactions at the extracellular end that locks drMfsd2a in the inward-facing state (Supplementary Movie 1).

### Rotation of the LPC headgroup

During the transport cycle, as lipids traverse from the outer to the inner leaflet, the substrates must translocate and also invert. As such, it is expected that both the acyl tail and headgroup of lipid-LPC will rotate during lipid flipping and translocation through Mfsd2a. Tracing the relative positions between Lysolipid_1A_ and Lysolipid_1B_ illustrate a significant rotation of the LPC headgroup (Figs. 1d, e, 2, Supplementary Fig. 12a, b). Unlike previous studies that suggest a linear substrate tunnel, our drMfsd2a structures revealed three distinct compartments that each comprise a separate hydrophobic pocket and a charged cavity to house the lipid tail and LPC headgroup (Figs. 2–3). We term the three hydrophobic pockets Chambers_1-3_ and the three charged cavities the zwitterionic traps_A-C_ (Z_A-C_) (Figs. 2–3, Supplementary discussion). Lysolipid_1A_, with the lipid tail in Chamber_1_ and LPC in Z_A_, is the closest substrate to the extracellular side and, hence, likely the first of the four ligand states captured during the substrate transport in our drMfsd2a structures (Figs. 1c–e, 2a, b). Chamber_1_, also observed in the chicken Mfsd2a structure^19^, is more extensive here in drMfsd2a (Fig. 2a, b, Supplementary discussion). All the residues in Chamber_1_ are highly conserved (Supplementary Fig. 6) and mutations at F59, M181, L308, F312, I333, F396, and W400 reduce lysolipid transport by Mfsd2a^19,29^ (Table 1). Based on their placement in Chamber_1_ and Z_A_, the lipid tail and headgroup of Lysolipid_1A_ are both pointed outwards, bent at an ~70° angle (Figs. 1e, 2a, b. Supplementary Fig. 12a). This suggest that lysolipid_1A_ is an intermediate state with lipid tail already rotated to align with the inner membrane leaflet but a headgroup that is yet to be flipped.

**Table 1:** Transport activities of Mfsd2a mutants

Residue	Mutation	Transport activity	drMfsd2a site	DrMfsd2a lysolipid	drMfsd2a function
Tyr50	Ala^29^	Reduced	Z_A_	Lysolipid_1A_	H-bond
Gln51	Leu^19^	Reduced	Z_A-B_	Lysolipid_1A_	H-bond
Phe59	Ala^29^	Reduced	Chamber_1_	Lysolipid_1A, 1B_	Lipid tail interaction
Arg84	Ala/Lys/Glu^19^	Abolished	Z_A_	Lysolipid_1A, 1B_	Salt-bridge
Asp87	Ala^29^	Abolished	Z_A_	Lysolipid_1A_	Salt-bridge
His156	Ala^29^	Abolished	Z_B_	Lysolipid_2B_	H-bond
Ser160	Leu^26,29^	Reduced	Z_B_	Lysolipid_2B_	H-bond
Arg180	Ala^29^	Abolished	Z_B_	Lysolipid_1B, 2B_	Salt-bridge
Met181	Ala/Phe^19^	Reduced	Chamber_2_	Lysolipid_2B_	Lipid tail interaction
Thr182	Phe/Met^29^	Reduced	Chamber_3_	Lysolipid_3c_	Lipid tail interaction
Glu184	Ala^29^	Reduced	Z_A-B_	Lysolipid_1A, 1B_	H-bond
Thr188	Phe^29^	Reduced	Chamber_1-2_	Lysolipid_1A, 1B, 2B_	Lipid tail interaction
Phe298	Ala^29^	Reduced	Chamber_1_	Lysolipid_1A, 1B_	Lipid tail interaction
Leu308	Trp^19^	Reduced	Chamber_1_	Lysolipid_1A, 1B_	Lipid tail interaction
Phe312	Ala/Tyr^19^	Reduced	Chamber_1_	Lysolipid_1A, 1B_	Lipid tail interaction
Ile333	Ala^19^	Reduced	Chamber_1_	Lysolipid_1A, 1B_	Lipid tail interaction
Ile341	Trp^19^	Abolished	Chamber_2-3_	Lysolipid_2B, 3C_	Lipid tail interaction
Gln345	Trp^19^	Reduced	Z_C_	Lysolipid_3C_	H-bond
Ala388	Trp^19^	Reduced	Chamber_1_	Lysolipid_1A, 1B_	Lipid tail interaction
Met334	Ala^19^	Reduced	Chamber_1-2_	Lysolipid_1A, 1B, 2B_	Lipid tail interaction
Phe396	Ala/Ile/Trp^19^	Reduced	Chamber_1-2_	Lysolipid_1A, 1B, 2B_	Gating, Lipid tail interaction
Trp400	Ala/Phe^19^	Reduced	Chamber_1-3_	Lysolipid_1-3_	Gating, Lipid tail interaction
Glu421	Arg^33^	Reduced	Z_A_	Lysolipid_1B, 2B, 3C_	Salt-bridge
Thr432	Ala^29^	Increased	Z_A-B_	Lysolipid_1A_	H-bond
Lys433	Ala^29^, Gln^19^	Abolished/Reduced	Z_A_	Lysolipid_1A_	Salt-bridge

Previous studies suggest that Mfsd2a transports lipid-LPC in a Na^+^ dependent manner^6,33^. It was proposed that the Mfsd2a symporter uses the flow of Na^+^ down its electrochemical gradient, from the outside to the inside of the cell, to drive the conformational changes for the transport and flipping of lysolipids. Molecular dynamics and functional analysis of the chicken Mfsd2a proposed a conserved and dynamic Na^+^ binding site annotated as D87, D91, E309, and K433 (drMfsd2a sequence numbers)^19^. Similar Na^+^ binding sites are also proposed in the mouse and human Mfsd2a structures^29,30^. These studies, however, do not provide clear structural details about how Na^+^ orchestrates lysolipid transport and flipping, thus require further investigation. In the Lysolipid_1A_ structure, the LPC headgroup is placed adjacent to the previously proposed Na^+^ binding location^6,19,33^ (Fig. 2a). Although we are not able to confidently place Na^+^ at this resolution, the placement of the LPC of lysolipid_1A_ into the elongated lipid-like density adjacent to the previously proposed Na^+^ site^6,19,33^ could provide important insights into the Na^+^-coupled transport mechanism by Mfsd2a. The colocalization of the headgroup and the cation suggest possible direct interaction of the Na^+^ with LPC, perhaps with the phosphate. In the lysolipid_1A_ configuration, the zwitterion phosphate and choline moieties are rigidified by (1) salt-bridges to D87, R84 and K433, (2) hydrogen bonds to Y50, Q51, and T432, and (3) electrostatic interactions with Q149 and Q152 (Fig. 2b, Supplementary Fig. 12a). This LPC binding arrangement is supported by studies illustrating that Mfsd2a can only transport lysolipids with headgroups that comprise of (1) a negative charge, but not necessarily a phosphate, and (2) an amine moiety^20,33^. We term the Mfsd2a interactions with the Lysolipid_1A_ LPC as zwitterion trap_A_ (Z_A_). Consistent with these observations, Y50, Q51, D87, R84, T432, and K433 are highly conserved (Supplementary Fig. 6) and their mutations alters or prohibit Mfsd2a lysolipid transport^19,29^ (Table 1).

In the second elongated density fitted with Lysolipid_1B_, we observed a lipid tail that is also tethered in Chamber_1_ but the LPC headgroup is now translocated by 16.9 Å, rotated by ~70° inwards, bound by a second zwitterion trap, Z_B_ (Figs. 1e, 2a, c, Supplementary Fig. 12b). The Z_B_ site that coordinates the LPC of Lysolipid_1B_ is comprised of (1) two salt-bridges by R180 and E421 to the phosphate and choline moieties and (2) is further rigidified by hydrogen bonding to M181, W400, and Y425 (Fig. 2c, Supplementary Fig. 12b). This LPC binding arrangement in Z_B_, again, supports the necessity of a headgroup that contains a negatively charged moiety and an amine group for lipid transport by Mfsd2a^20,33^. All residues that comprise Z_B_ coordination sites are conserved (Supplementary Fig. 6) and mutations of Q51, M181, R180, W400, and E421 reduce Mfsd2a lipid-LPC transport activity^19,29,33^ (Table 1). Unlike the LPC headgroup, the translocation of C18 of the lipid tail of Lysolipid_1B_ from Lysolipid_1A_ is more modest and still adhered in Chamber_1_ (Figs. 1a-c, 2a-c). Due to the shift in Lysolipid_1B_, Chamber_1_ is modified for binding the lipid tail with fewer hydrophobic interactions (Fig. 2c, Supplementary discussion). Together, the trajectory from Lysolipid_1A_ to Lysolipid_1B_ describes a ~70° rotation and 16.9 Å translocation of the LPC from a head-out to a head-in orientation (Figs. 1d, e, 2, Supplementary Fig. 12a–b). In contrast to Lysolipid_1A_, which is only partially rotated, both the lipid tail and LPC headgroup of lysolipid_1B_ have been flipped to align with the inner membrane leaflet.

### Lipid translocation

After the rotation of the LPC headgroup, we propose that the next steps during lysolipid transport involve the translocation of the flipped lipid-LPC by Mfsd2a towards the cytoplasmic side (Fig. 3). In the Lysolipid_2B_ configuration, the C18 of the ALA lipid tail is displaced 13.9 Å relative to Lysolipid_1B_ (Fig. 1a, c, e) and is bound in Chamber_2_ located towards the cytoplasmic side of drMfsd2a (Fig. 3a, b). Chamber_2_ is shifted inward from Chamber_1_ and is comprised of highly conserved residues (Fig. 3b, Supplementary Fig. 6, Supplementary discussion) and mutation of M181, T188, and I341 disrupt or abolish Mfsd2a transport activity^19,29^ (Table 1). Unlike the large movement in the lipid tail, the LPC of Lysolipid_2B_ is bound in a similar position to Lysolipid_1B_ by Z_B_ (Figs. 2a, 3a). The phosphate and choline of Lysolipid_2B_ are also held in place by salt-bridges to R180 and E421 but is now shifted more towards the cytoplasmic side, forming hydrogen bonds with H156 (via a water), S160, and E184 (Fig. 3a, b, Supplementary Fig. 12c). Consistent with our findings, H156, S160, R180, E184, and E421 are conserved (Supplementary Fig. 6). Mutation of H156, S160, R180, E184, and E421 disrupt or abolish Mfsd2a transport activity^19,26,29^ (Table 1). It is noteworthy that mutation of S160 is associated with decreased DHA transport to the brain and results in severe microcephaly^26^.

Examination of Lysolipid_3C_ revealed that the lipid tail is in a third hydrophobic chamber, Chamber_3_, located adjacent to the cytoplasmic opening of the drMfsd2a substrate tunnel (Fig. 3a, c). Comparison with Lysolipid_2B_ indicates that the C18 of the lipid tail and choline of Lysolipid_3C_ have translocated inwards by 8.1 and 4.9 Å, respectively, (Figs. 1c–e, 3a). The more modest sized Chamber_3_ is exposed at the opening of the cytoplasmic side of the drMfsd2a substrate tunnel (Figs. 1e, 3a, c, Supplementary discussion). All residues that comprise Chamber_3_ are highly conserved (Supplementary Fig. 6) and mutation of I341 and W400 limit or abolish Mfsd2a transport activity^19^ (Table 1). Similar to the fewer interactions that bind the lipid tail, the LPC of Lysolipid_3C_ is coordinated by a single salt-bridge of the choline by E421 and two hydrogen bonds to D174 and Q345 (Fig. 3c, Supplementary Fig. 12d). Consistent with these observations, D174, Q345, and E421 are highly conserved (Supplementary Fig. 6) and mutation of the latter two decrease or prohibit Mfsd2a lysolipid transport activity^19,33^ (Table 1). After lipid rotation, the transition from Lysolipid_1B_ to Lysolipid_3C_ describes a 22 Å and 9.4 Å translocation of the C18 of the lipid tail and the choline of the headgroup from the center towards the cytoplasmic side of the drMfsd2a substrate tunnel (Fig. 1c–e).

### Open and closed conformations of the Mfsd2a gates

It has been proposed that substrate “gating” of Mfsd2a is comprised of bulky hydrophobic residues that respond to the presence of and orchestrate the conformational changes required for transport of lysolipids^19,34^. Molecular dynamics simulations of the chicken Mfsd2a proposed “inner gates” that are located within the hydrophobic tunnel consisting of the conserved residues M181, F396, and W400^19^ (Supplementary Fig. 6). Indeed, we observe movements of F396 and W400 but not M181 that is dependent on the presence of a nearby acyl-chain (Fig. 3d, e). In the Mfsd2a substrate tunnel, F396 and W400 interact with lipid tails in Chamber_1-2_ and Chamber_1-3_, respectively (Figs. 2–3). In the structures with the lipid tail absent from Chamber_2_, F396 and W400 are flexible, occupying multiple conformations that block access to Chamber_2_ (Fig. 3d). In the presence of lipid tail of Lysolipid_2B_ in the Chamber_2_, F396 and W400 occupy a single conformation that allow access to the substrate tunnel (Fig. 3e). Here, F396 and W400 are now also engaged in forming van der Waals interactions with the acyl-chain of the Lysolipid_2B_ within Chamber_2_ (Fig. 3e). We propose that the “gating” interactions with these bulky hydrophobic residues facilitate the translocation of the lipid tail of lipid-LPC from Chamber_1_ through Chamber_3_. Consistent with the above postulate, functional analyses coupled with mutagenesis of F396 and W400 reduce Mfsd2a lipid transport^19^ (Table 1). Moreover, molecular dynamics simulations indicated similar findings where the presence of Na^+^ and substrate cargo stimulated the opening of the F396 and W400 gates^19^ to allow lipid tail access to Chamber_2_.

### Lipid release and inner membrane integration

Based on its unique position, the following features observed in our studies suggest Lysolipid_3C_ is poised for release from Mfsd2a for integration into the inner membrane leaflet. First, there are fewer contacts between the lipid tail and drMfsd2a of Chamber_3_ than Chamber_1-2_ (Figs. 2–3, Supplementary discussion). Second, there are also minimal contacts between LPC of Lysolipid_3C_ and Z_C_ (Fig. 3c, Supplementary Fig. 12d). Third, Lysolipid_3C_ is placed in a groove at the opening of the drMfsd2a substrate tunnel that is exposed on the cytoplasmic side of the lipid bilayer (Figs. 1c–e, 3a, c, 3f). Fourth, when mapped to the adjacent DDM molecules, Lysolipid_3C_ is closely aligned with the detergent belt that encircles the cytoplasmic side of drMfsd2a, likely resembling where the inner membrane leaflet would interact with the transporter (Fig. 3f). Given these rationales, we propose that Lysolipid_3C_ is poised for release through the cytoplasmic opening of the substrate tunnel of Mfsd2a for integration into the inner membrane leaflet (Figs. 1e, 3f).

### Proposed lipid flipping and Mfsd2a cycling

**Fig. 4:**
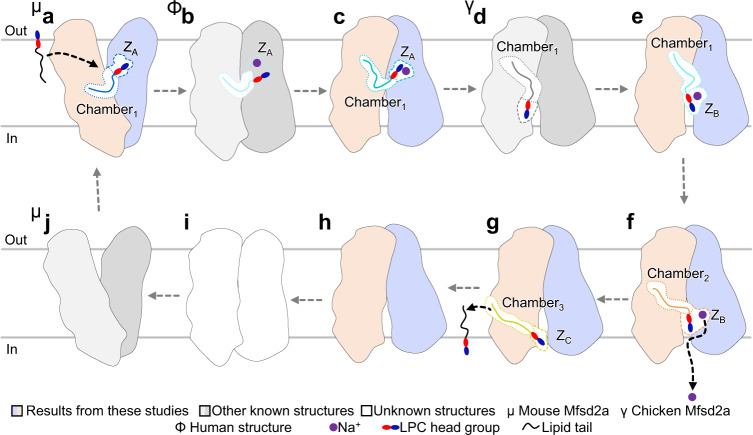
Proposed model for Mfsd2a lipid transport cycle. **a** The ligand-bound, outward-facing mouse Mfsd2a structure docked with ALA-LPC is bound in a lateral position Chamber_1_ (see Supplementary discussion). The LPC headgroup is trapped by Z_A_ in an outward pointing orientation. **b** The proposed and unknown occluded ligand-bound conformation. Mfsd2a rocks from an outward to inward-facing conformation. **c**–**e** The various conformations during flipping of the LPC headgroup. The lipid tail is held in position by Chamber_1_ while the LPC headgroup samples multiple configurations between Z_A_ and Z_B_ before it is moved to Z_B_. This movement flips the LPC headgroup from an outward to inward-pointing orientation. This process results in a substrate with the lipid tail pointing out and the LPC headgroup pointing in, a ligand configuration that is aligned with the inner membrane leaflet. **f** The translocation of the lysolipid to Chamber_2_ and Z_B_. The lipid tail is translocated from Chamber_1_ to Chamber_2_. The LPC headgroup is shifted inwards. This process translocates the entire lipid-LPC substrate closer to the cytoplasmic exit. **g** The release of the substrate to the cytoplasmic side. LPC headgroup is moved to Z_C_ and the lipid tail is translocated to Chamber_3_. Chamber_3_ and Z_C_ are located at the cytoplasmic exit. **h** The substrate-free structure of drMfsd2a in the inward-facing conformation. This structure represents the conformation after lysolipid release from Mfsd2a. **i**–**j** The conformations during resetting of the transporter. In the absence of the substrate, Mfsd2a resets to the outward-open conformation (**j**) by first transitioning through a ligand-free occluded state (**i**).

During the transport of the substrate, flippases perform the energetically unfavorable task of flipping the lipids from the outer to the inner membrane leaflet (Fig. 4). To our knowledge, a structural model fully detailing a lipid flipping mechanism has not been elucidated for flippases, particularly for the MFS system. The most well studied lipid flipping system is the P4-ATPase flippase. There are several nucleotide-dependent structures of P4-ATPases with alternate conformations of the soluble domains, however, with only one lipid bound on the extracellular side^35^. More recently, there is now a P4-ATPase flippase structure bound with the lipid already flipped with the headgroup towards the cytoplasmic side^36^. The missing gap is how the ATP-dependent structural changes of the soluble domains promote lipid rotation within the P4-ATPase transmembrane region. Further, P4-ATPase and Mfsd2a flippases are different structurally and one uses ATP and the other a Na^+^ gradient to drive lipid rotation and transport. As such and while it would provide important comparative analysis, these two flippase systems likely employ different mechanisms for lipid flipping and transport. Here, using the five structures determined, together with the previously determined structures of Mfsd2a, we propose that lipid flipping occurs in a series of finely orchestrated steps by which the lipid tail and LPC headgroup are interchangeably anchored by the transporter through interactions with Chambers_1-3_ and Z_A-C_ that are separated into three distinct compartments as the phospholipid traverses through the substrate tunnel (Figs. 2–4).

Since the lipid-LPC translocates from the outer to the inner membrane leaflet, the first anchoring event observed in this study is the transition between Lysolipid_1A_ to Lysolipid_1B_ where the lipid tail is tethered to Chamber_1_ while the headgroup is free to flip and sample the open cavity from Z_A_ to Z_B_ (Figs. 2, 4c–e, Supplementary Movie 3). Once the headgroup is attached to Z_B_, as seen for Lysolipid_1B_ (Figs. 2c, 4e), the tail is released from Chamber_1_ and transferred to Chamber_2_ where Lysolipid_2B_ is found (Figs. 3b, 4f). While the positions of the four lipid-LPC span the length of the drMfsd2a substrate tunnel (Fig. 1e), key steps are still missing. Namely, it is unclear whether the lipid tail or LPC headgroup anchors first to Chamber_3_ or Z_3_ (Figs. 3, 4f, g). Our structures do, however, demonstrate that the lysolipid exits Mfsd2a through an opening of the substrate tunnel on the cytoplasmic side (Figs. 1e, 3f, 4g, Supplementary Movie 3c).

In addition to the substrate, the roles of sodium in lipid transport and flipping remain elusive. It is still unclear whether sodium is involved directly in lipid flipping, however, the close proximity of the LPC of lysolipid_1A_ to the proposed Na^+^ binding site suggests possible contacts between the cation and the headgroup (Fig. 2a). If true, it is tempting to suggest a model of the coupled movement of Na^+^ with lipid-LPC down the electrochemical gradient of the cation during translocation and flipping of the lysolipid through Mfsd2a (Figs. 2–3, 4c–f, Supplementary Movie 3). While the cryo-EM maps presented here do display a density at this site, it is unclear at this resolution whether there is indeed a sodium bound. As such, further studies are required to clarify the involvement of the sodium, or lack of, in the lipid flipping mechanism. Together, the lipid tail versus LPC anchoring model provides a mechanistic model where the lipid flips and translocate in a stepwise manner through three separate compartments until it emerges out at the apposing end and inserts into the inner membrane leaflet.

## Discussion

In this study, five structures of *dr*Mfsd2a were described, including four unique lipid sites. The structures allow us to propose a stepwise lipid flipping mechanism of Mfsd2a. Extensive mutagenesis and functional analyses confirm the importance of key residues identified for lysolipid transport and flipping. While these snapshots allow us to propose a stepwise lipid flipping mechanism, several unanswered questions still remain. For example, is sodium necessary for lipid flipping? If yes, where is the sodium bound? Is this transport mechanism conserved for the various lipid-LPC that can be transported by Mfsd2a? When does acyl-chain inversion occur? Here, we have preliminary docking simulations that suggest that acyl-chain inversion happens before the rotation of the headgroup and before the transition of Mfsd2a from the outward to inward-facing conformation (Supplementary Fig. 13). As such, future studies should further investigate if and how the change in protein conformation, specifically the transition from outward to inward-facing positions, affect lipid flipping and transport. Along the same line, is the protein simply acting as a scaffold for lipid flipping or is the rocker-switch critical in ushering the lipid through this process? To provide a more complete understanding, future studies must delve more deeply into the proposed flippase and lipid transport for Mfsd2a and for other less common lipids that play critical roles in neuromodulation.

## Methods

### Protein expression and purification

Mfsd2a isoform B from zebrafish (Uniprot Q6DEJ6) was subcloned in the pFasbac1 vector (Invitrogen) with an N-terminal-10xHis tag. The Mfsd2a sequence was truncated at residue D22 of the N-terminus, D509 of the C-terminus, and with the N509Q, N214Q, N225Q mutations to remove post-translational glycosylation modifications. DH10bac was used for preparation of bacmids. Recombinant baculovirus was generated using the *Spodoptera frugiperda* Sf-9 system and transfection following the protocol provided for the Bac-to-bac Baculovirus Expression System. Mfsd2a-pFasbac1 construct was used to transfect into Sf-9 cells (ThermoFisher, 12659017) following a standard protocol to generate three generations of baculovirus. P3 generation of the Mfsd2a-pFasbac1 baculovirus was used to infect a batch culture of Sf-9 for protein expression.

Mfsd2a was overexpressed in Sf-9 insect cells, harvested 60 h after infection. The cell pellets were resuspended in lysis buffer containing 20 mM Tris (pH 8.0) and 150 mM NaCl and supplemented with EDTA-free protease inhibitor cocktail (Roche). The Sf-9 cells were lysed using 50 homogenizing cycles on ice. The lysate was clarified by centrifugation at 130,000 × *g* for 1 h. The pelleted membrane was resuspended in high-salt buffer containing 1.6 M NaCl and 20 mM Tris (pH 8.0) for washing and centrifuged again for 1 h at 130,000 × *g* to remove soluble debris. The pelleted membrane was rapidly frozen in liquid nitrogen and stored at –80 °C until further use.

To purify Mfsd2a, the membrane pellet was solubilized and membrane protein extracted in 2% n-dodecyl-β-d-maltopyranoside (DDM), 20 mM Tris (pH 8.0), 150 mM NaCl, 5% glycerol and 0.2% cholesteryl hemisuccinate Tris salt (CHS) for 4 h at 4 °C. After extraction, the insoluble debris were removed by centrifugation for 1 h at 130,000 × *g*. 20 mM imidazole (pH 8.2) was added to the supernatant and incubated with TALON beads for 16 h at 4 °C. The Mfsd2a-bound beads were washed with 6 column volumes of 20 mM imidazole, 20 mM Tris (pH 8.0), 500 mM NaCl and 0.1% DDM. The resin was then equilibrated in buffer composed of 20 mM Tris (pH 8.0), 150 mM NaCl, 0.4% decyl-β-d-maltoside (DM), and 0.02% DDM. At 4 °C, the 10 × His tag was removed by on-column TEV digestion overnight at an enzyme:protein molar ratio of 1:500. The cleaved Mfsd2a was collected in the flow-through and was flash frozen in liquid nitrogen and stored at −80 °C until further use.

### FAB production and purification

Mouse IgG monoclonal antibodies against drMfsd2a were produced by the Monoclonal Antibody Core (D. Cawley). 330 µg of purified Mfsd2a in buffer containing 20 mM Tris (pH 8.0), 150 mM NaCl, 0.02% DDM and 0.002% CHS was used to immunize mice in three injections. 15 × 96-well plate fusions yielded 169 IgG-positive wells at a 1:30 dilution. Native and denatured Mfsd2a proteins were then used in ELISA to search for candidates that bound the conformational epitopes27, where Ni-NTA plates were used for Mfsd2a immobilization. Thirty-five of the 169 fusions showed significant preference for binding against well-folded Mfsd2a. Western blotting was performed to assess the binding affinity and specificity of the antibodies generated from hybridoma cell lines. Monoclonal antibody 11D3 was then purified from the hybridoma supernatants by 4-mercaptoethylpyridine (MEP) chromatography. FAB fragments were produced by papain digestion and purified in flow-through buffer containing 20 mM NaPi (pH 8.0) and 150 mM NaCl by protein A affinity chromatography.

### Mfsd2a-FAB complex assembly

Purified 15A9 FAB fragment was incubated overnight at 4 °C with Mfsd2a at a 5:1 molar ratio. Mfsd2a–15A9 complex was injected into the size exclusion column Superdex200 to separate the unbound FAB. The size exclusion column step was also used to exchange the buffer of the drMfsd2a-FAB complex into 50 mM HEPES (pH 8.0), 150 mM NaCl and 0.02% DDM. SDS-PAGE analysis was used to pool peak fractions containing both Mfsd2a and FAB, indicating complex formation (Supplementary Fig. 1). The pooled fractions containing Mfsd2a-FAB were pooled and concentrated to 3 mg/ml using an Amicon spin concentrator with a 30 kDa cutoff.

### Mfsd2a-FAB Cryo-EM grid preparation

400-mesh 1.2/1.3 Cu or 300-mesh UltrAuFoil 1.2/1.3 Au grids (Quantifoil) were made hydrophilic by glow discharging for two times 60 seconds with a current of 15 mA in a PELCO easiGlow system. The cryo grids were prepared using a Leica EM GP (Leica). The chamber was kept at  4 °C and 95% humidity (86–91% measured). Three microliters of sample at 3 mg/ml were applied to a glow-discharged holey grids, blotted for 6 s, and plunge frozen into liquid ethane and stored in liquid nitrogen.

### Mfsd2a-FAB cryo-EM data collection

Cryo-EM grids were loaded into a 300 keV FEI Titan Krios cryo-electron microscope (ThermoFisher Scientific, formerly FEI) at HHMI Janelia Reasearch Campus, Janelia Krios 1, equipped with a C_s_ corrector, and Gatan energy filter and K3 camera (Gatan Inc.). Movies of 50 frames with 1 e^−^/Å^2^ per frame (50 e^−^/Å^2^ total dose) were automatically recorded at a nominal magnification of ×81,000, corresponding to a physical pixel size of 0.844 Å/px (superresolution pixel size 0.422 Å/px) in CDS mode at a dose rate of 9.5 e^−^/px/s (~7.5 e−/px/s on the camera through the sample) and a defocus range of −0.5 to −1.8 µm using SerialEM^37^. In total, 2653 and 8443 movies were collected in two separate imaging sessions, with the first dataset on a 400-mesh copper grid and the second on a 300-mesh UltrAuFoil gold grid (Table S1).

### Mfsd2a-FAB cryo-EM data processing

The overall workflow of the image processing is illustrated in Supplementary Fig. 2. All preliminary steps were performed within RELION 3.1^38^ unless specified. Movie alignment and dose weighting were performed with MotionCor2^39^ in a 5 × 5 patch. Contrast transfer function (CTF) parameters were estimated with Gctf^40^. Good 2D classes generated from ~1000 manually picked particles served as templates for automatic particle picking in RELION, resulting in 623,644 and 3,033,688 particles in dataset 1 and dataset 2, respectively. Particle images extracted were 4× binned, resulting in a pixel size of 3.376 Å, and then subjected to several rounds of 2D classification and 3D classification, removing junk particles and particles without FAB bound. Particles were re-extracted, unbinned to a pixel size of 0.844 Å, and subjected to 3D auto-refinement with local angular searches (RELION additional arguments: --sigma_ang 1.667) followed by Bayesian polishing. Polished particles from 2 datasets were imported into cryoSPARC v3.3.2^41^. For dataset 1, 146,914 particles were subjected to CTF refinement to correct for beam-tilt, spherical aberrations, and per-particle defocus parameters, followed by non-uniform refinement resulting in a map at 3.3 Å. For dataset 2, 346,394 particles were first cleaned up by heterogenous refinement, followed by CTF refinement and non-uniform refinement resulting in a map at 3.2 Å. Two datasets were merged and further classified by heterogenous refinement and refined to 2.9 Å with local resolution regions up to 2.4 Å using non-uniform refinement and local resolution filtering. Altogether, more than 60 maps (from cryoSPARC) between 2.9 and 4.13 Å were created using different strategies of processing the data using cisTEM^42^, RELION^38^, and cryoSPARC^41^, showing different degrees of details, especially around the ligand binding sites.

Densities for three ligands in the 2.9 Å map from 295,580 particles were clearly observed in the cavities of Mfsd2a which are unlikely to co-exist simultaneously. We hypothesized that the three ligand densities shown in that particular density map are a result of merged particle populations where subsets with different ligand positions have not been classified due to the small ligand size. To sort out subsets with different ligand positions, 3D variability analysis^43^ and 3D classification without alignment using a tight mask covering the ligand binding sites were used without obvious success. Alternatively, reference-based 3D classification without alignment was deployed. Individual ligand densities were segmented and extracted with UCSF Chimera^44^. Extracted ligand densities were then subtracted from the Mfsd2a-FAB map, generating references with (1) 3 ligands bound, (2) no ligand-bound, (3) only ligand 1 bound, (4) only ligand 2 bound, and (5) only ligand 3 bound, respectively. These maps served as references for 3D classification without alignment, with a tight mask covering ligand binding sites included. To avoid reference bias and overfitting, individual particle subsets obtained from 3D classification were subjected to ab-initio reconstruction, followed by non-uniform refinement, with initial model lowpass-filtered to 30 Å, resulting in maps at 3.3 Å, 3.4 Å, 3.3 Å, 3.4 Å, and 3.4 Å, with local resolutions to up to 2.8 Å (3 ligands, lower resolution), 2.8 Å (ligand-free), 2.8 Å ( A), 2.9 Å (2B), and 2.9 Å (3C), respectively (Supplementary Fig. 2, Supplementary Table 1). To ensure model bias was not inherited throughout the workflow after classification without alignment, a control experiment was performed. A Mfsd2a-FAB map and a map with TM1 segment subtracted were used for classification without alignment, followed by ab-initio reconstruction and non-uniform refinement (Supplementary Fig. 3). The results showed that the TM1 density was clearly observed in both classes and the maps after refinement of each subclass, demonstrating reference bias was mitigated throughout the workflow applied in the study. In addition, we imported the particles of the individual subclasses from cryoSPARC into RELION. 3D refinement of the individual subclass with an initial model lowpass-filtered to 40 Å yielded maps at 3.8–4.1 Å, which also showed individual ligand density. The map with the clearest Lysolipid_1B_ configuration was generated from 413,435 particles using a combination of processing in cisTEM^42^ and cryoSPARC^41^ and has an average resolution of 4.1 Å (Table S1). The local resolution of all maps was additionally estimated using RELION’s own implementation where half maps and masks from cryoSPARC were imported as inputs (Table S1). Six of the >60 maps have been made available and have the following accession codes: EMD-27148, EMD-27149, EMD-27150, EMD-27151, EMD-27152, and EMD-27153.

### Model building and refinement

The initial model fitting was achieved by manual rigid body rotation and translation and the “Fit Map” of the chicken Mfsd2a structure^19^ (PDB 7MJS) into the 2.9 Å electron density maps for drMfsd2a in UCSF Chimera^44^. The protein sequence of the structures was altered to match drMfsd2a and manually fitted into the maps in Coot^45^. Building of the FAB portion of the structure was achieve similar to drMfsd2a starting with the FAB model from the SLC38A9-FAB^46^ structure (PDB 6C08). Refinement of the drMfsd2a structure was accomplished by iterative cycles of manual flexible fitting and automatic refinement in Coot^45^ and Real Space Refine in Phenix^47,48^, respectively. The final structure validation was carried out in Real Space Refine in Phenix^48^ (Table S1). The fitted pdbs have been made available together with the EM maps with the following PDB IDs: 8D2S, 8D2T, 8D2U, 8D2V, 8D2W, and 8D2X. Figures and movies were generated in Pymol or UCSF Chimera^44^.

### In vitro endogenous lipid analysis

Unsaturated free fatty-acids were detected and quantified using the Lipid Assay kit (abcam ab242305). Purified drMfsd2a was concentrated to 15 mg/mL and used for lipid quantification according to manufacturer’s instructions. Briefly, 15 μL of samples were dispensed in tubes and incubated at 90 °C for 30 min, then 4 °C for 5 min. 150 μL of 18 M sulfuric acid was added to each sample. Samples were then incubated for 10 min at 90 °C, then 5 min at 4 °C. One hundred microliters of each sample were transferred to a 96-well plate and mixed with 100 μL of vanillin and incubated at 37 °C for 15 min. Absorbance was measured at OD 560 nm. Data reported are mol-to-mol ratio of three technical replicates calculated using molecular weights 517.6 g/mol for lipids and 56.6 kDa for drMfsd2a. The standard curve for phospholipid quantification was generated using the standards from the Lipid Assay Kit.

### Proteoliposome reconstitution

Chloroform-dissolved chicken egg phosphatidylcholine (egg-PC, Avanti Polar Lipids) was evaporated using dry nitrogen to yield a thin lipid film in a small glass vial and further desiccated under vacuum overnight. The lipids were hydrated in inside buffer (20 mM Tris (pH 8.0), 150 mM KCl, 5 mM NaCl) at 25 mg/mL by vortexing for 3 min and then aged at room temperature for 1 h. Liposomes were clarified by 5 rounds of freezing and thawing in liquid nitrogen and extruded through a 100 nm membrane filter (Millipore) with 21 passes. The liposomes were pre-incubated with 1% n-octyl-β-D-glucoside (β-OG) and 1 mM DDT for 1 h at 4 °C before protein reconstitution. Purified Mfsd2a protein was incorporated at 1:100 (w/w) ratio into destabilized liposomes for 1 h in a 4 °C rotator. Five percent glycerol-supplemented protein buffer was used in lieu of Mfsd2a protein in liposome-only control groups. The detergents were removed by incubation overnight with 250 mg per reaction of Bio-Beads, and the proteoliposomes were further incubated with 50 mg per reaction of fresh Bio-Beads for an additional hour. The proteoliposomes and liposomes-only controls were collected using ultracentrifuge at 100,000 × *g* for 30 min at 4 °C and then resuspended in outside buffer (20 mM Tris (pH 8.0), 150 mM NaCl, 5 mM KCl) to a final lipid concentration of 32 μg/μL.

### Proteoliposome [^14^C]DHA-LPC uptake assay

Transport reactions were initiated by adding 0.1 μCi [^14^C]DHA-LPC (American Radiolabeled Chemicals, Inc) to 30 μL aliquots of proteoliposomes to a final concentration of 60 μM [^14^C]DHA-LPC. Assay of Liposome-only controls were carried out in parallel to experimental groups as negative controls. All buffers were chilled, and assays were performed at 30 °C in a metal bath.

For the steady-state uptake assay, after 10 min incubation, proteoliposomes were filtered, washed by 5 mL of ice-cold outside buffer, and collected on 0.22 μm nitrocellulose membrane filters (Millipore) which had been pre-wet with washing buffer (PBS pH 8.0). After washing, each filter was dried by vacuum for exactly 1 min and transferred into a glass vial with 10 mL scintillation fluid for counting. Non-specific adsorptions of [^14^C]DHA-LPC by liposomes-only controls were shown as control groups. The experiment was repeated three times and figures show the results with S.E.M error bar.

### Computational analysis of ALA-LPC versus DDM binding

For binding energy comparisons between the lysolipid and DDM modeled in each of the liganded states, pdb coordinates were prepared in MOE (Molecular Operating Environment) with the default simulation forcefield Amber10:EHT with R-Field solvation. QuickPrep-Protonate3D is used to prepare the PDB coordinates for adding hydrogens and assigning protonation states. Atom constraints were applied to the coordinates to fix the modeled positions in the electron density maps. Ligand properties were calculated after energy minimization for the chosen ligands.

### Molecular docking for ALA-LPC in the outward-open conformation of mouse Mfsd2a

The molecular docking of ALA-LPC in the outward-open conformation of mouse Mfsd2a^31^ (PDB 7N98) was calculated with the Chimera Autodock Vina tool^47^. The docking was performed within the defined bounding box in the extracellular access of the transmembrane domain, which included a volume of ~30,000 A^3^ as the search space. Ten docking poses were generated with exhaustiveness of search set at 8, maximum energy difference (kcal/mol) set at 2. The resulting poses yielded scores ranging from 6.2 to −5.7 kcal/mol. The best one was chosen following biochemical rationales.

### Reporting summary

Further information on research design is available in the Nature Portfolio Reporting Summary linked to this article.

## Supplementary information

Supplementary Information

Peer Review File

Description of Additional Supplementary Files

Supplementary Movie 1

Supplementary Movie 2

Supplementary Movie 3

Reporting Summary

## Data Availability

The data that support this study are available from the corresponding authors upon request. Cryo-EM maps have been deposited in the Electron Microscopy Data Base (EMDB) under accession codes EMD-27148 (Merged Lysolipid_1A,2B,3C_), EMD-27149 (Ligand-free), EMD-27150 (Lysolipid_1A_), EMD-27151 (Lysolipid_1B_), EMD-27152 (Lysolipid_2B_), and EMD-27153 (Lysolipid_3C_). The atomic coordinates have been deposited in the Protein Data Bank (PDB) under accession codes 8D2S (Merged Lysolipid_1A,2B,3C_), 8D2T (Ligand-free), 8D2U (Lysolipid_1A_), 8D2V (Lysolipid_1B_), 8D2W (Lysolipid_2B_), and 8D2X (Lysolipid_3C_).
