# Supplementary material for: Lipid flipping in the omega-3 fatty-acid transporter
Source: Nat Commun. 2023 May 8;14:2571. doi: 10.1038/s41467-023-37702-7 (PMC10167227; doi:10.1038/s41467-023-37702-7)
Supplement: Supplementary file 1 — Supplementary Information [file 41467_2023_37702_MOESM1_ESM.pdf]

Supplementary Information

Lipid flipping in the omega-3 fatty-acid transporter

Chi Nguyen[*1,2], Hsiang-Ting Lei[*1,3], Louis Tung Faat Lai[*4], Marc J. Gallenito[1,2], Xuelang Mu[1,2,5], Doreen Matthies[#3,4], Tamir Gonen[#1,2,5,6]

[1] Howard Hughes Medical Institute, University of California Los Angeles, Los Angeles, CA 90095
[2] Department of Biological Chemistry, University of California Los Angeles, Los Angeles, CA 90095
[3] Janelia Research Campus, Howard Hughes Medical Institute, 19700 Helix Drive, Ashburn, VA 20147
[4] Unit on Structural Biology, Division of Basic and Translational Biophysics, *Eunice Kennedy Shriver* National Institute of Child Health and Human Development, National Institutes of Health, Bethesda, MD 20892
[5] Molecular Biology Institute, University of California, Los Angeles, Los Angeles, CA 90095
[6] Departments of Physiology, University of California Los Angeles, Los Angeles, CA 90095

[*] These authors contributed equally

[#]correspondence addressed to D.M. doreen.matthies@nih.gov or T.G. tgonen@g.ucla.edu

Supplementary discussion
- Lipid tail-drMfsd2a interactions
- A proposed model for lipid flipping and Mfsd2a cycling

Supplementary figures and legends
- Supplementary Fig. 1. Assembly of the drMfsd2a-FAB complex
- Supplementary Fig. 2. Single-particle cryo-EM data processing for *dr*Mfsd2a-FAB complex
- Supplementary Fig. 3. Control experiment of the classification workflow with a TM1 truncated reference
- Supplementary Fig. 4. Fit of the *dr*Mfsd2a model with cryo-EM density
- Supplementary Fig. 5. ALA-LPC modeling into lipid-like densities
- Supplementary Fig. 6. Mfsd2a sequence alignment
- Supplementary Fig. 7. Comparison between the mouse, chicken, human, and drMfsd2a structures
- Supplementary Fig. 8. DrMfsd2a endogenous lipid analysis
- Supplementary Fig. 9. In vitro proteoliposome reconstitution and [^{14}C]DHA-LPC uptake assay
- Supplementary Fig. 10. Computational analysis of ALA-LPC versus DDM and DM binding
- Supplementary Fig. 11. Detergent fit into lipid-like density
- Supplementary Fig. 12. The four LPC binding configurations in drMfsd2a
- Supplementary Fig. 13. Docking studies of ALA-LPC in the outward-facing mouse Mfsd2a structure

Supplementary videos

Supplementary Video 1. Interdomain contacts and rocker-switch movement between the inward and outward conformation of Mfsd2a

Supplementary Video 2. Transition between the outward, outward-occluded, and inward Mfsd2a structures

Supplementary Video 3. Moving figures of protein conformational changes and lipid flipping, translocation, and release by drMfsd2a

Supplementary tables

Supplementary Table 1. Cryo-EM data collection, refinement, and validation statistics

Supplementary Table 2. Ligand binding analysis and maps used

Supplementary discussion

Lipid tail-drMfsd2a interactions

The interactions between drMfsd2aFS2A and the lipid tails were identified by visual inspection of residues that surround the acyl-chain for each ALA-LPC in Coot. These interactions are listed as below and illustrated in Figures 2-3.

$Chamber_1$ - $Lysolipid_{1A}$: Residues F59, M181, V185, T188, F305, M306, L308, F312, F315, V329, L330, I333, M334, A337, V364, F367, L368, V371, A384, A388, V392, F396, W400, Y428, and V471 (Fig. 2a-b).

$Chamber_1$ - $Lysolipid_{1B}$: Residues F59, V185, T188, F298, F305, M306, L308, F312, V329, L330, I333, M334, A337, V364, L368, A388, V392, A395, F396, W400, and Y428 (Figure 2a, c).

$Chamber_2$ - $Lysolipid_{2B}$: Residues M181, V185, T188, L189, M334, A337, T338, I341, V392, A393, F396, L397, and W400 (Figure 3a-b).

$Chamber_3$ - $Lysolipid_{3C}$: A178, T182, V185, L186, L189, L335, T338, L339, I341, L397, and W400 (Figure 3a, c).

A proposed model for lipid flipping and Mfsd2a cycling

To gain further insights into the initial binding and flipping of the lysolipid, we performed docking experiments of ALA-LPC on the outward-facing mouse Mfsd2a structure[29] (Supplementary Fig. 13). There are three key findings in our docking studies. First, the lipid tail is bound in a lateral orientation, dictated by the shape of a modified $Chamber_1$ that faces outwards towards the extracellular side (Fig. 4a, Supplementary Fig. 13). Second, we observe that the docked LPC is bound in the same cavity that comprise residues of Z_A (Fig. 2, 4a). Third, similar to our $Lysolipid_{1A}$, the LPC is pointed outward, an orientation of the lysolipid that is still aligned with the outer leaflet (Fig. 4a, Supplementary Fig. 13). We believe these docking studies represent a lysolipid that is yet to be flipped and bound in the transporter before the transitions from an outward to an inward-facing conformation (Fig. 4a-b, Supplementary Fig. 13). Therefore, we propose that during the inward rocking motion, $Chamber_1$ changes from an outward-facing, lateral position to an inward, vertical conformation (Fig. 2a, 4a-c, Supplementary Fig. 13). If true, the change in conformation of $Chamber_1$ to a vertical position is likely driving the force to flip the lipid tail from a lateral to a vertical, outward pointing orientation, as observed for $Lysolipid_{1A}$ (Fig. 1e, 2a-b, 4c, Supplementary Video 3). Moreover, because the headgroup of $Lysolipid_{1A}$ is still in Z_A after Mfsd2a changes from the outward to inward-facing conformation, the flipping of the acyl-chain outwards results in a bent $Lysolipid_{1A}$ where both the lipid tail and LPC are pointing outwards (Fig. 1e, 2a-b, 4c, Supplementary Video 3). Given these results, we propose that the inversion of the acyl-chain takes place before the headgroup. Specifically, we propose that the reorientation of the lipid tail to point outwards occurs during the transition of Mfsd2a from the outward to inward-facing conformation and is the first key step in the flipping of the lysolipid to align with the inner membrane leaflet (Fig. 4a-c, Supplementary Video 3).

After the reorientation of the acyl-chain to point outwards to align with the inner membrane leaflet, the largest rotation of the headgroup occurs between the transition from $Lysolipid_{1A}$ to $Lysolipid_{1B}$ (Fig. 4c-e). The flipping of the LPC from Z_A to Z_B appears to be

facilitated by the following features. First, there is an open cavity between Z_A to Z_B, allowing delocalized LPC movements between the two sites zwitterion traps (Fig. 2a). This is consistent with our observation of weaker density for the LPC versus the lipid tail for Lysolipid$_{1A}$ (Fig. 1e, Supplementary Fig. 5a) and the double conformation seen for the headgroup in the chicken Mfsd2a structure[19]. Second, the flipping of the LPC from Z_A to Z_B is possible by stabilization of the lipid tail in Chamber$_1$ while the headgroup samples multiple binding sites in the open cavity between Z_A and Z_B (Fig. 2a). Because the lipid tail is rigidified in Chamber$_1$ and the LPC is translocated from Z_A and eventually trapped in Z_B, the headgroup can reorient from the outward, bent Lysolipid$_{1A}$ to a more linear inward pointing configuration, as observed in Lysolipid$_{1B}$ (Fig. 2, 4c-e). Given these observations, the rotation of the headgroup from the Z_A-bound Lysolipid$_{1A}$ to the Z_B-bound Lysolipid$_{1B}$ is the next key step in lysolipid flipping. During the process, the headgroup the lysolipid is flipped from the outward to inward-pointing orientation to align the lipid-LPC to the inner membrane leaflet (Fig. 4c-e). Therefore, we propose that the reorientation of the LPC to point the headgroup inward occurs after rotation of the lipid tail outwards and is the next key step in flipping the lysolipid to align with the inner membrane leaflet.

Supplementary figures and legends

Supplementary Fig. 1. Assembly of the drMfsd2a-FAB complex. a-b, Size exclusion chromatography (a) and SDS-PAGE analysis (b) of drMfsd2a-FAB complex formation. Protein was eluted in 0.5 ml fractions. Fractions collected for each sample are indicated. These experiments were repeated independently four times yielding the same results.

Supplementary Fig. 2. Single-particle cryo-EM data processing for drMfsd2a-FAB complex. a, Workflow of cryo-EM image processing of drMfsd2a-FAB. The resolution was reported according to the Fourier shell correlation (FSC) = 0.143 criteria. **b,** A representative cryo-EM micrograph of drMfsd2a-FAB and 2D class averages with a box size of 162 Å. Over 10,000 electron micrographs were collected on two separate occasions displaying particles of similar morphology and distribution. **c,** Local resolution evaluation of the drMfsd2a-FAB map at an average 2.9 Å resolution. **d-e,** Evaluation of the cryo-EM reconstruction of the drMfsd2a-FAB final maps with FSC curves (d) and Euler angle distribution plot of the merged lysolipid$_{1A,\ 2B,\ 3C}$ map. (e).

Supplementary Fig. 3. Control experiment of the classification workflow with a TM1 truncated reference. An intact map and a map with TM1 segment removed were used for reference-based 3D classification without alignment. The TM1 density was clearly observed in both classes and the maps after ab-initio reconstruction followed by refinement of each sub-class.

Supplementary Fig. 4. Fit of the drMfsd2a model with cryo-EM density. Cryo-EM densities (mesh) are superimposed on the TMs (**a**). The model is shown in stick representation.

Supplementary Fig. 5. ALA-LPC fitting into lipid-like densities. Alternate views of the modeling of lysolipid$_{1A}$ (**a**), lysolipid$_{1B}$ (**b**), lysolipid$_{2B}$ (**c**), and lysolipid$_{3C}$ (**d**) into the four lipid-like densities observe in drMfsd2a.

Supplementary Fig. 6. Mfsd2a sequence alignment. Clustal Omega sequence alignment between human (*H. sapiens*, UniProtKB - Q8NA29), zebrafish (*D. rerio*, UniProtKB - Q6DEJ6), chicken (*G. gallus*, (NCBI XM_417826), and mouse (*M. muscuslus*, UniProtKB - Q9DA75)

drMfsd2a. Loops and helices are indicated by lines and bars. Residues for Z-site binding, Z-site and lipid interacting, and lipid interacting residues are highlighted in green, wheat and gray, respectively. Unassigned N- and C-terminal domains in dotted line.

Supplementary Fig. 7. Comparison between the mouse, chicken, human, and zebrafish Mfsd2a structures. a, Alignment of mouse[31] (PDB 7n98) and drMfsd2a. Mouse Mfsd2a is in gray. drMfsd2a is in teal. **b,** Alignment of human[32] (PDB 7OIXoix) and *dr*Mfsd2a. Human MFSD2A is in light cyan. DrMfsd2a is in teal. **c,** Alignment of chicken[20] (PDB 7mjs) and drMfsd2a. Chicken Mfsd2a is in green. DrMfsd2a is in teal. ALA-LPC from chicken[20] Mfsd2a is in green stick and sphere representation. Lysolipid$_{1A}$ is in dark cyan stick and sphere representation.

Supplementary Fig. 8. DrMfsd2a endogenous lipid analysis. a, Standard curve for lipid detection assay. **b,** Total phospholipid concentration calculation. **c,** Calculation of phospholipid:protein ratio. **b-c,** The data presented are derived from the measurements of three independent samples and as mean values +/- SEM.

Supplementary Fig. 9. In vitro proteoliposome reconstitution and [^{14}C]DHA-LPC uptake assay. a, Reconstitution of drMfsd2a into liposomes. Protein concentration as compared to BSA standard. **b,** In vitro radiolabeled [^{14}C]DHA-LPC proteoliposome uptake assay. The data presented are derived from the assessment of three independent samples and as mean values +/- SEM.

Supplementary Fig. 10. Computational analysis of ALA-LPC versus DDM and DM binding. a, B-factors of modeled ALA-LPC versus DDM and DM at the four observed lipid-like densities. B-factors calculations were performed in Phenix Refinement. **b,** Energy calculations are from modeled ALA-LPC, DDM, and DM using MOE.

Supplementary Fig. 11. Detergent vs. lysolipid fit into lipid-like density. a, A detergent molecule (DDM) fit into the lipid-like density of Mfsd2a. Several clashes were observed as the hydrophilic headgroup of the detergent was forced into a hydrophobic pocket. This analysis is consistent with a lipid occupying the site **(b** Lysolipid$_{1A}$**)** rather than a detergent molecule. Protein side chains in dark gray stick.

Supplementary Fig. 12. The four LPC binding configurations in drMfsd2a. **a-d,** Alternate views of lysolipid$_{1A,\ 1B,\ 2C,\ \text{and}\ 3C}$ positions. Lysolipids are in stick and sphere representation. Z-sites coordinating residues are in stick. Black dotted lines represent H-bonding between 2.6-3.3 Å. Red dotted lines indicate salt bridges with distances ≤4 Å. Blue half circles indicate choline coordinating residues within 3.5 Å. Waters in red small spheres.

Supplementary Fig. 13. Docking studies of ALA-LPC in the outward-facing mouse Mfsd2a structure. **a-b,** The outward open mouse Mfsd2a structure[31] (PDB 7n98) with a docked ALA-LPC substrate shown as cartoon (a) and surface (b) representation. Docked ALA-LPC shown as blue stick and sphere.

Supplementary tables

Supplementary Table 1. Cryo-EM data collection, refinement, and validation statistics.

Supplementary Table 2. Ligand-drMfsd2a intermolecular interactions analysis and models used.

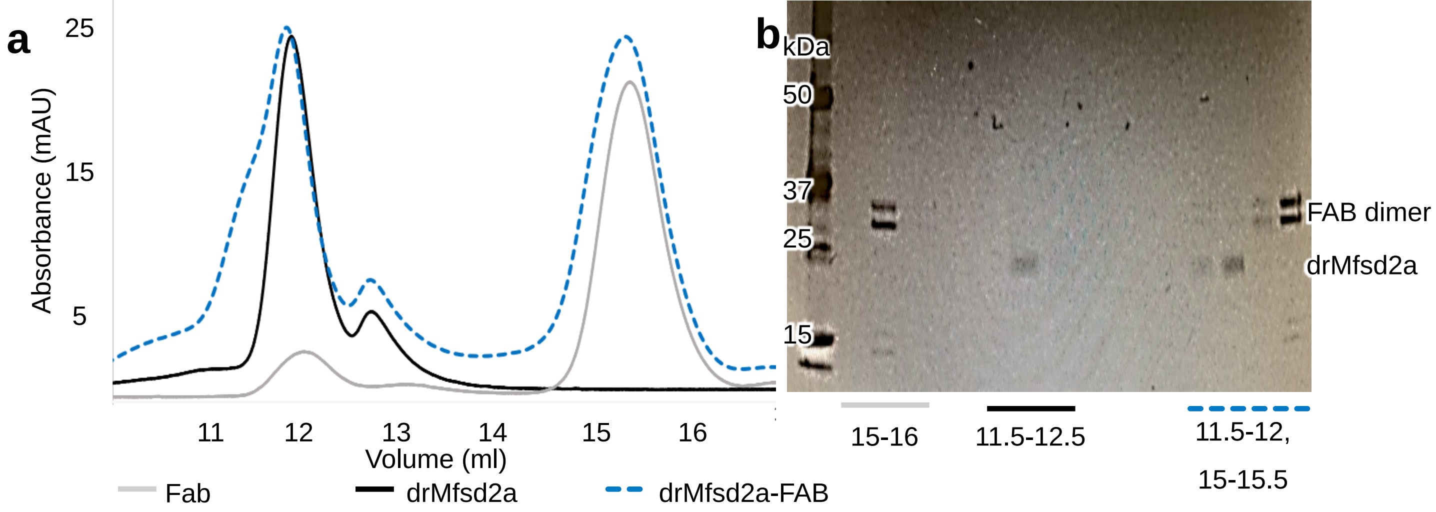

Supplementary Fig. 1

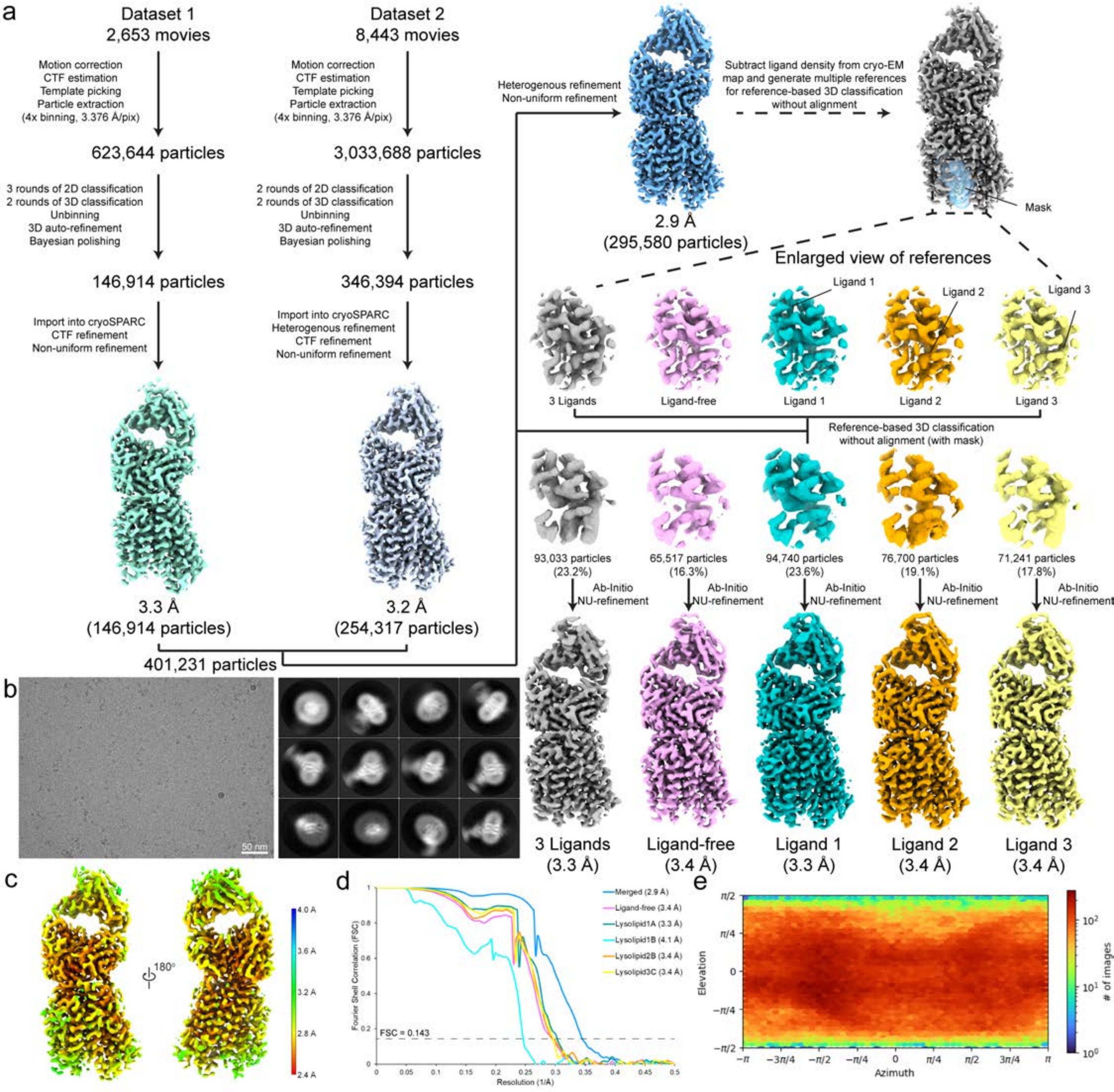

Supplementary Fig. 2

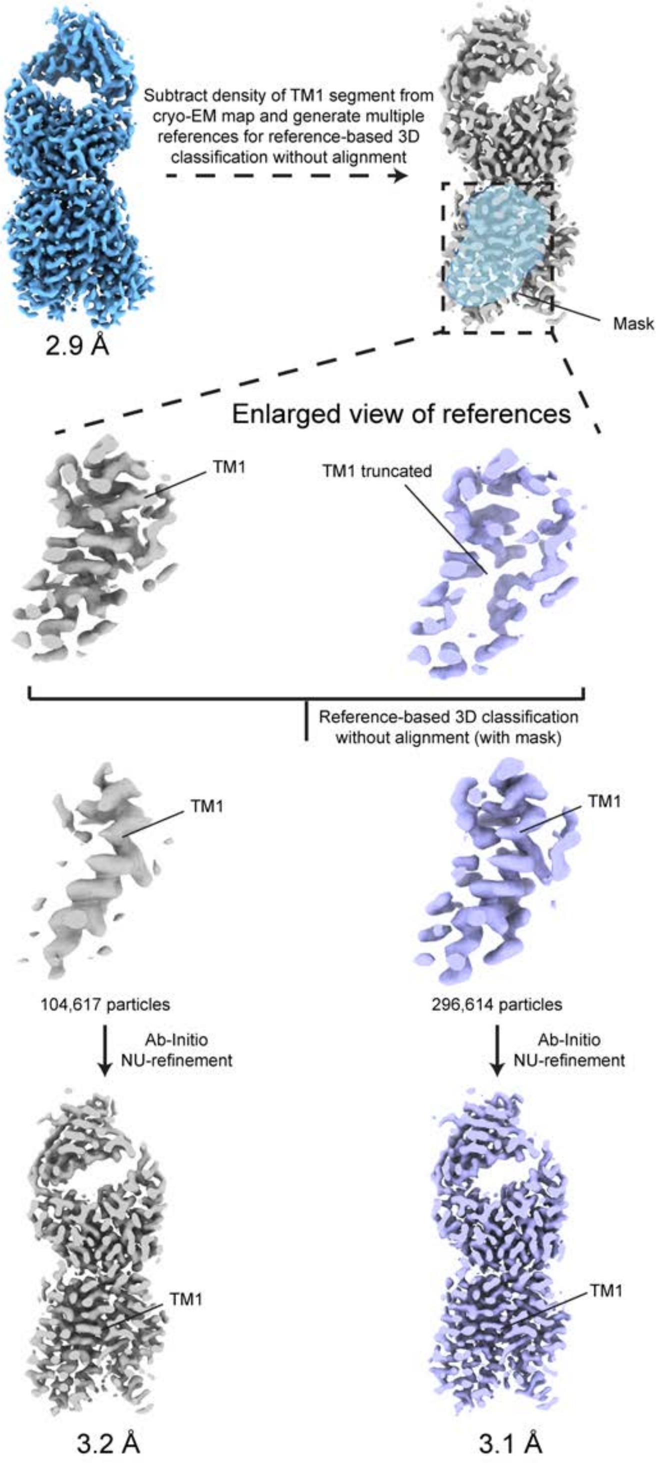

Supplementary Fig. 3

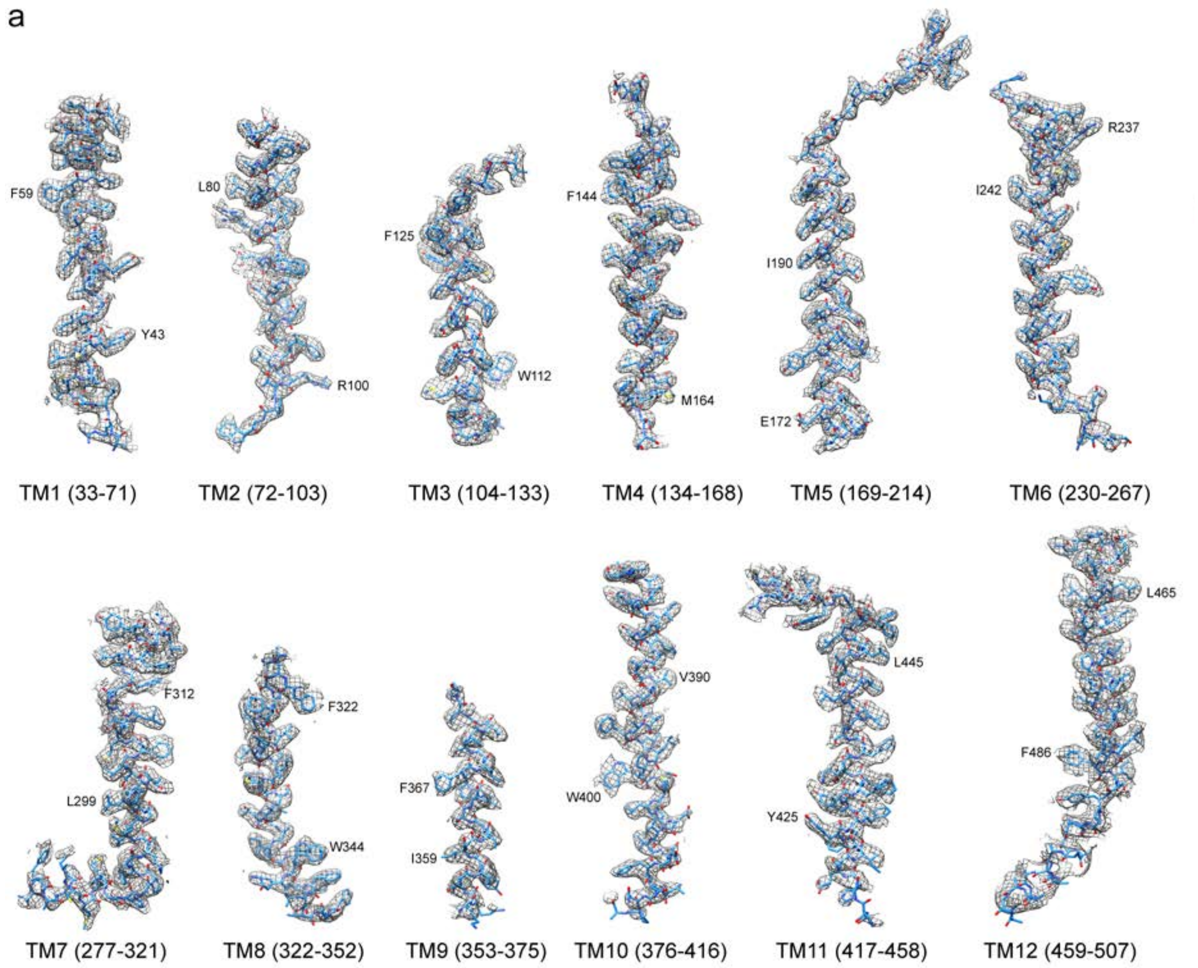

Supplementary Fig. 4

a

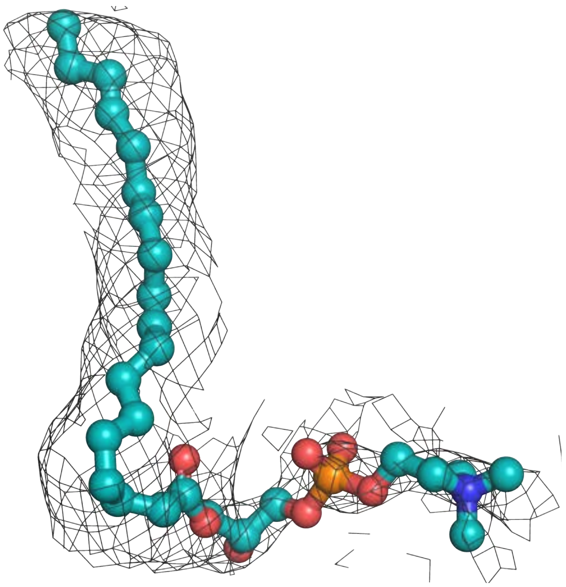

b

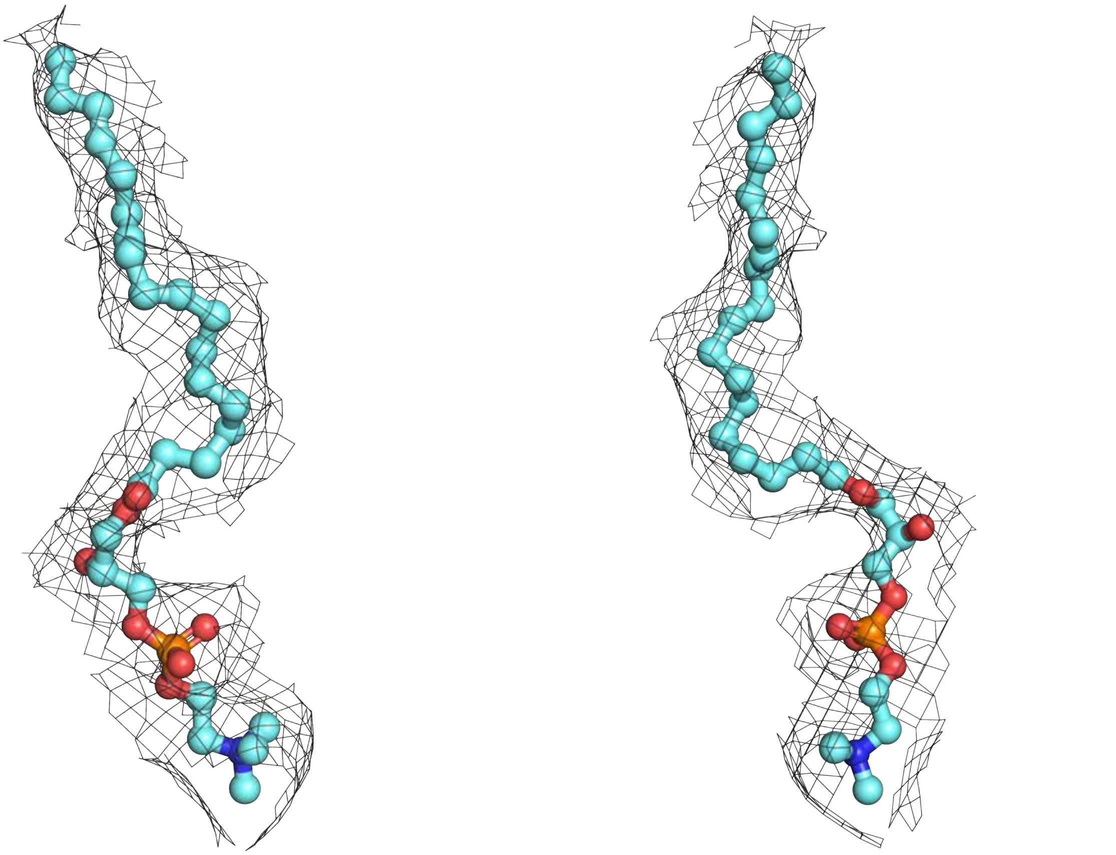

Supplementary Fig. 5 continue on next page

c

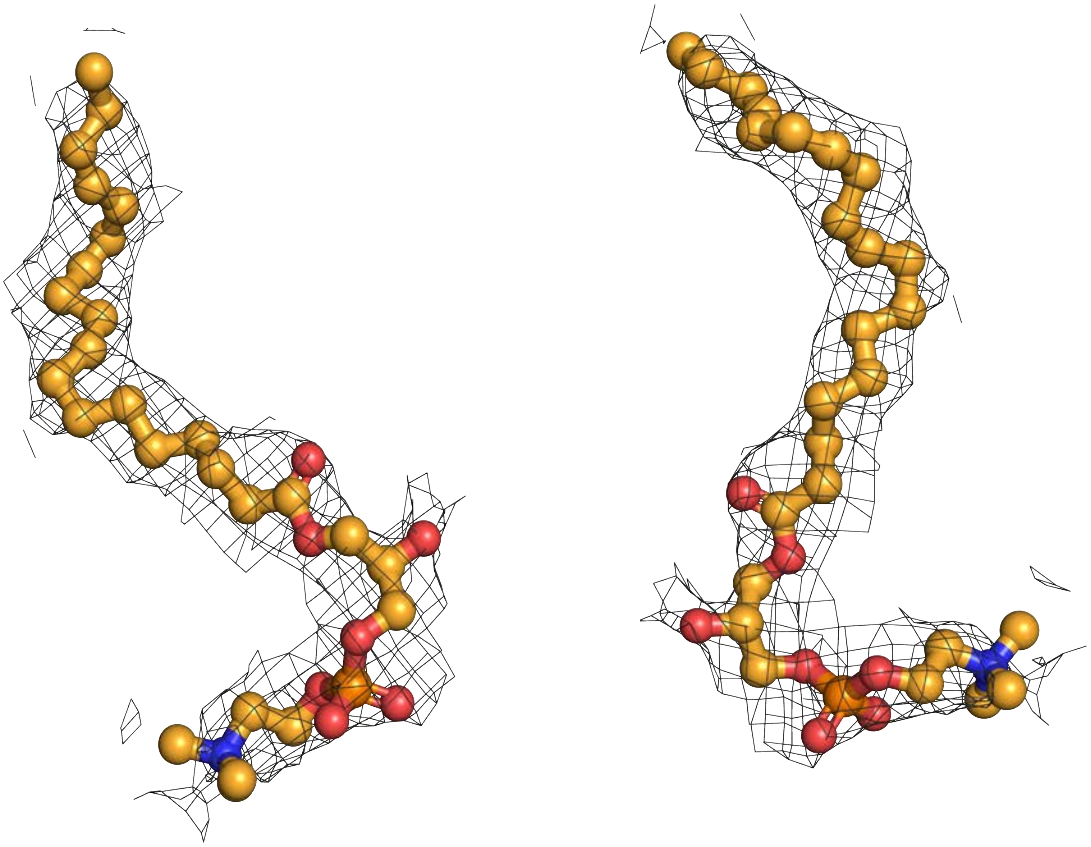

d

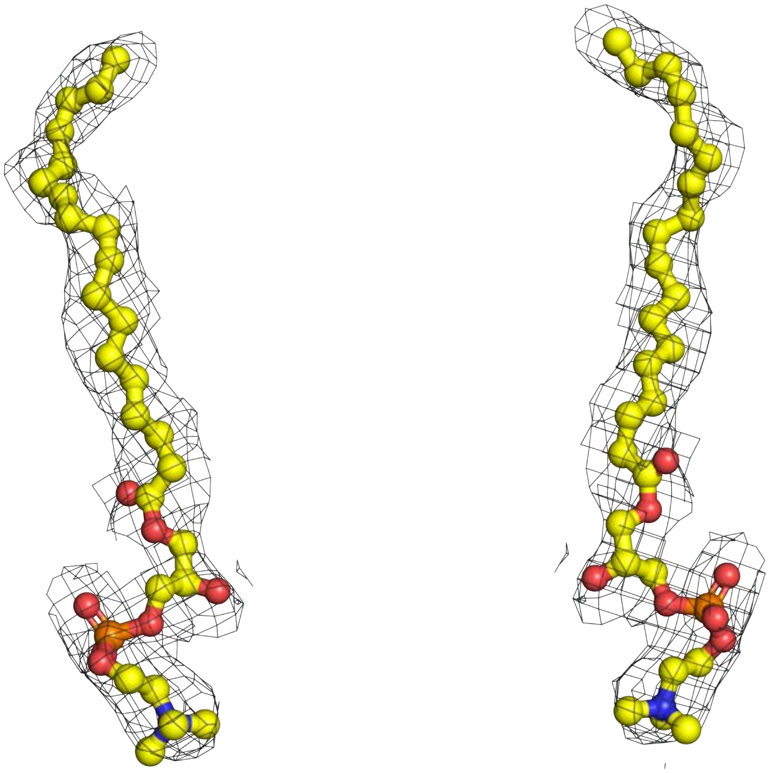

Supplementary Fig. 5

Supplementary Fig. 6

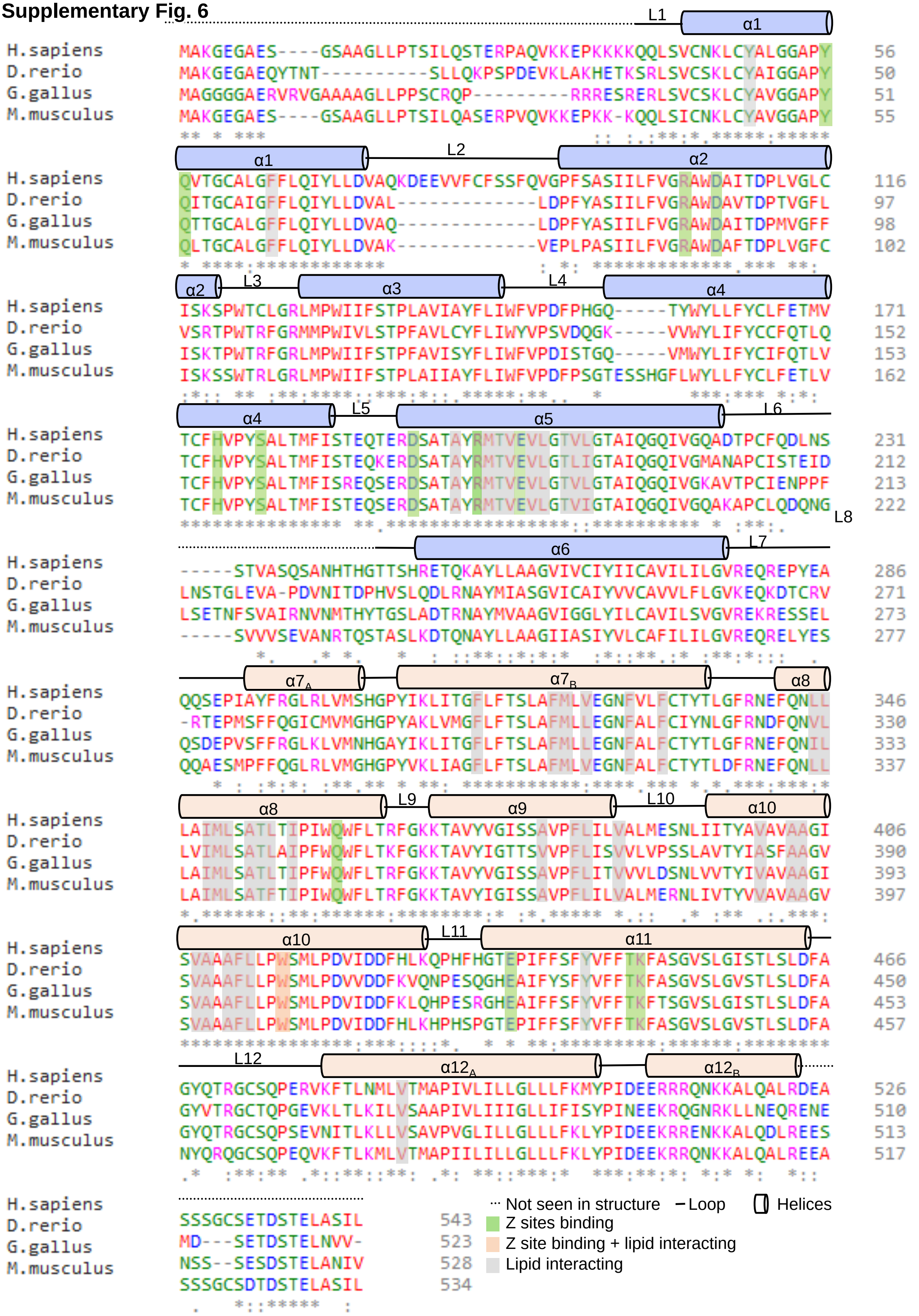

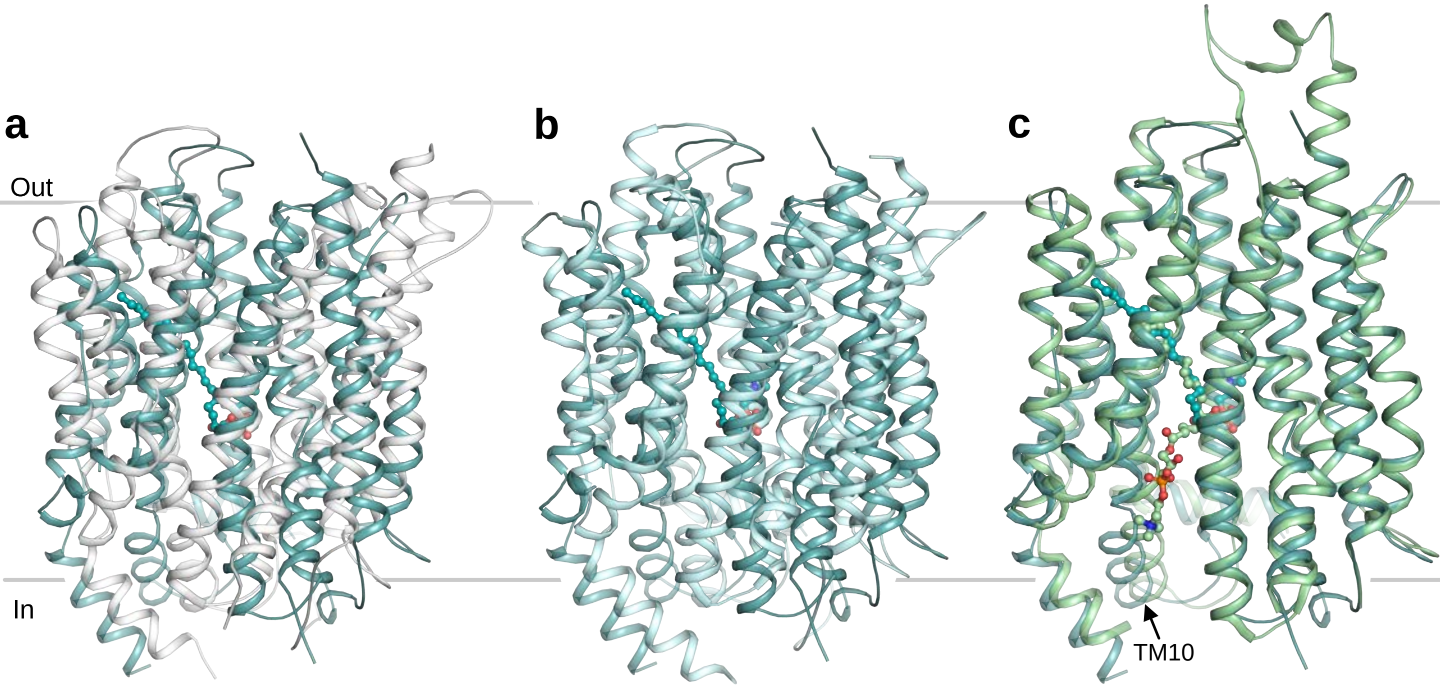

Supplementary Fig. 7

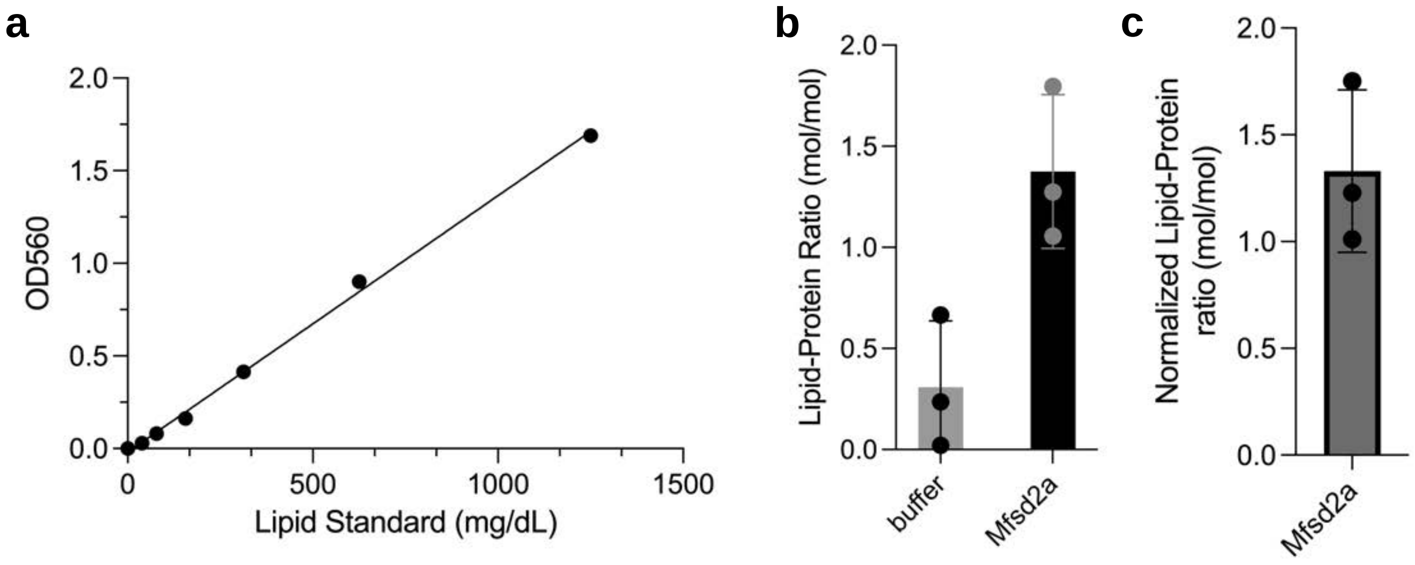

Supplementary Fig. 8

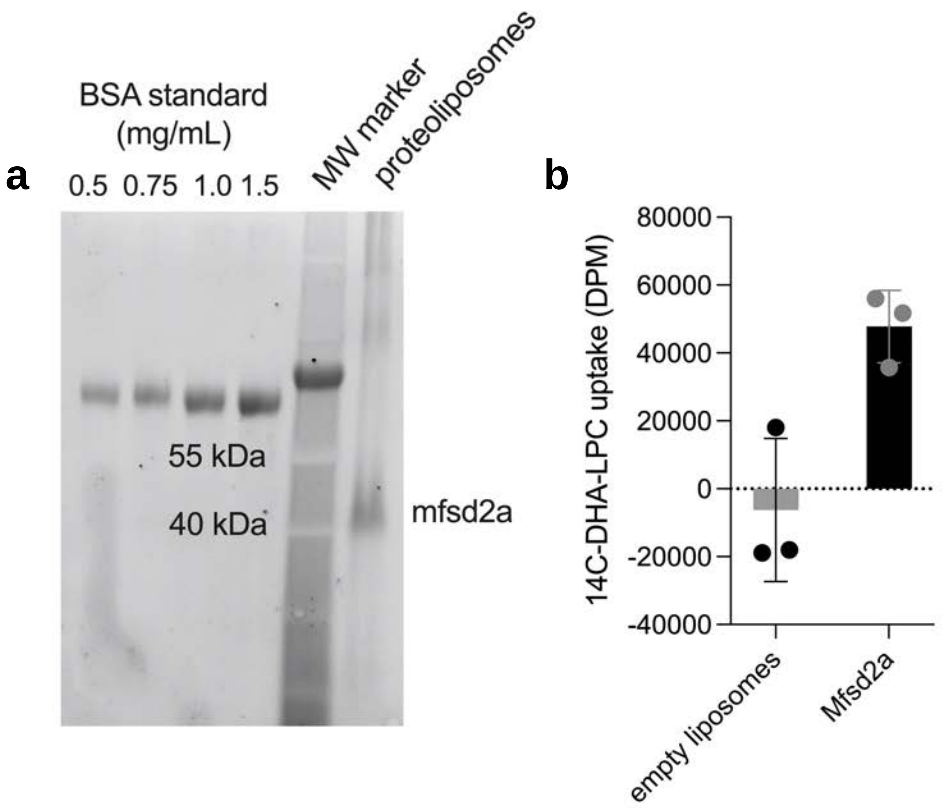

Supplementary Fig. 9

a

	ALA-LPC	DDM	DM
**1A**	34.0	43.4	34.1
**1B**	70.6	103.8	83.9
**2B**	34.2	60.9	37.7
**3C**	34.1	52.1	56.2

Values B-factor

b

	ALA-LPC	DDM	DM
**1A**	-7.5	-2.1	-2.0
**1B**	-7.3	-6.9	2.9E10
**2B**	-8.5	-7.8	1.6E6
**3C**	-6.7	-4.7	-4.8

Values represents energy (kcal/mol)

Supplementary Fig. 10

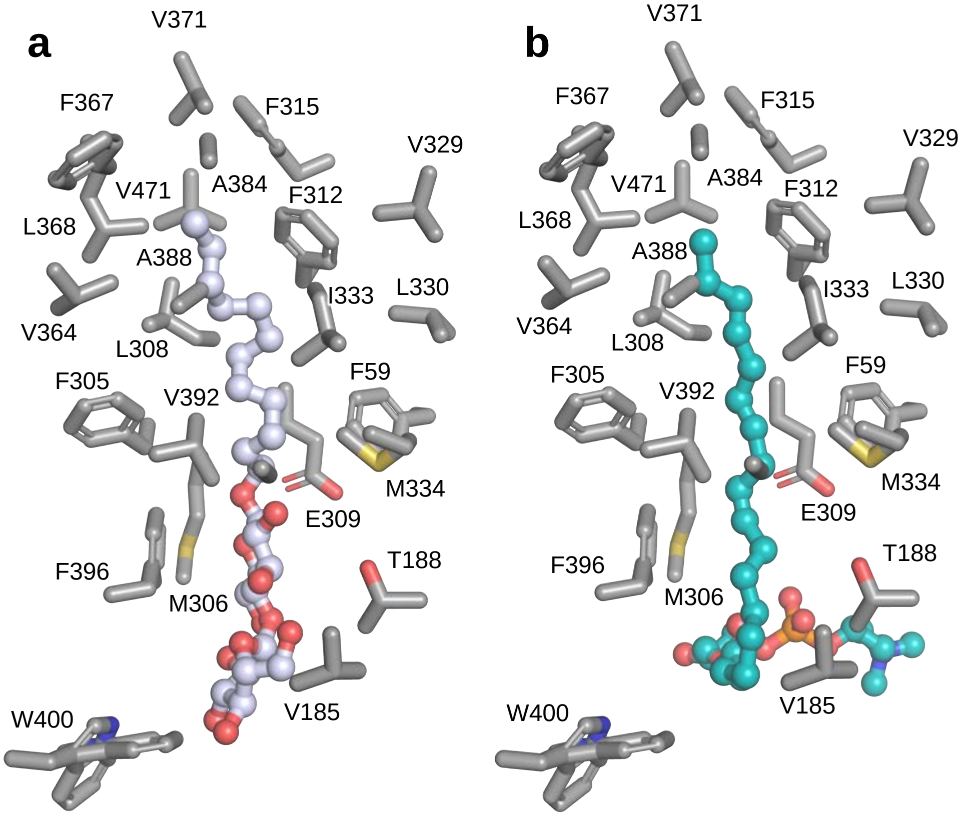

Supplementary Fig. 11

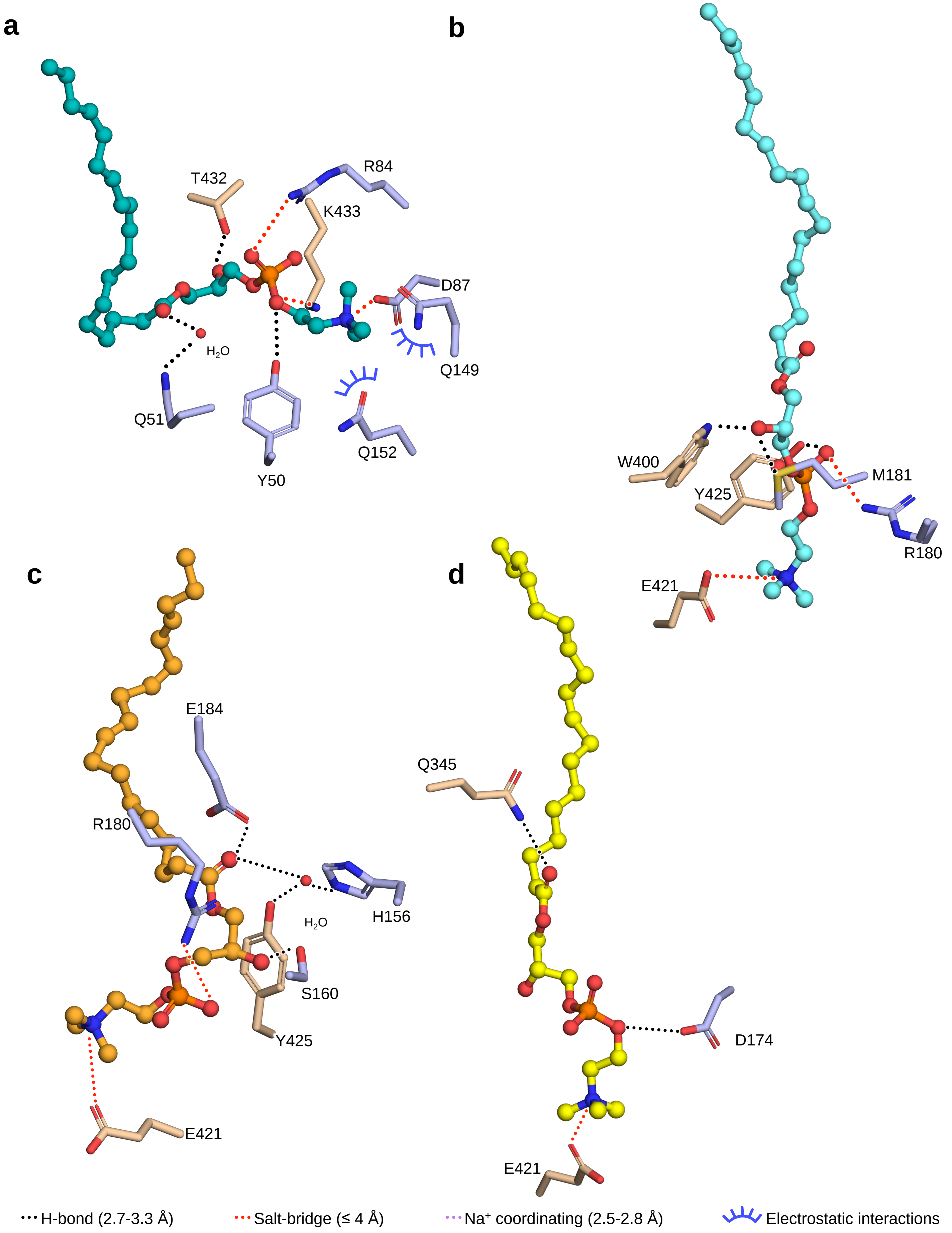

Supplementary Fig. 12

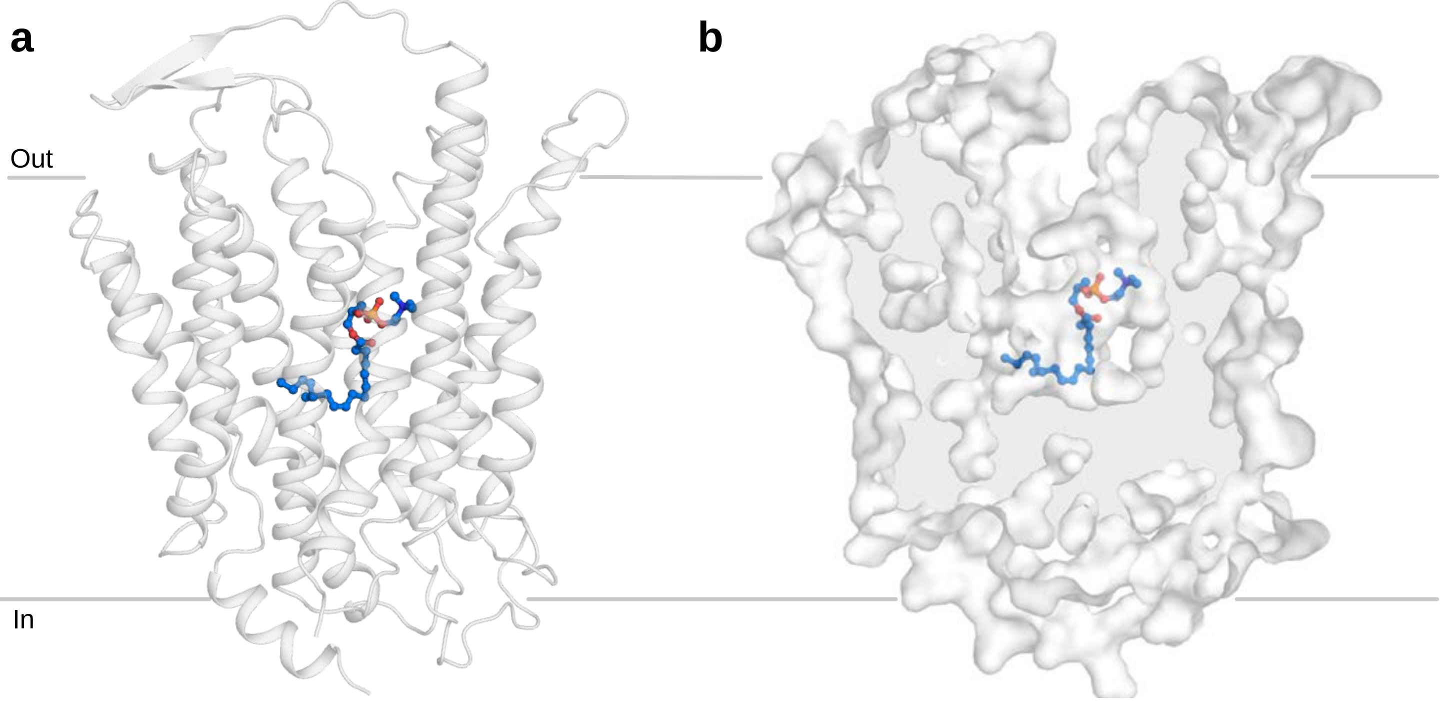

Supplementary Fig. 13

Supplementary Table 1

	Merged Lysolipid$_{1A, 2B, 3C}$ (EMDB-27148) (PDB 8D2S)	Ligand-free (EMDB-27149) (PDB 8D2T)	Lysolipid$_{1A}$ (EMDB-27150) (PDB 8D2U)	Lysolipid$_{1B}$ (EMDB-27151) (PDB 8D2V)	Lysolipid$_{2B}$ (EMDB-27152) (PDB 8D2W)	Lysolipid$_{3C}$ (EMDB-27153) (PDB 8D2X)
**Data collection and processing**						
Magnification	81,000	81,000	81,000	81,000	81,000	81,000
Voltage (kV)	300	300	300	300	300	300
Electron exposure (e^{-}/Å^2)	50	50	50	50	50	50
Defocus range (μm)	0.5-1.8	0.5-1.8	0.5-1.8	0.5-1.8	0.5-1.8	0.5-1.8
Pixel size (Å)	0.844	0.844	0.844	0.844	0.844	0.844
Symmetry imposed	C1	C1	C1	C1	C1	C1
Initial particle images (no.)	3,657,332	3,657,332	3,657,332	2,802,513	3,657,332	3,657,332
Final particle images (no.)	295,580	65,517	94,740	413,435	76,700	71,241
Map resolution (Å)	2.90	3.40	3.30	4.10	3.40	3.40
FSC threshold	0.143	0.143	0.143	0.143	0.143	0.143
Map resolution range (Å)	2.40-3.30	2.90-4.40	2.80-4.60	2.50-7.00	2.90-4.50	2.90-4.50
**Refinement**						
Initial model used (PDB codes)	7MJS, 6C08	7MJS, 6C08	7MJS, 6C08	7MJS, 6C08	7MJS, 6C08	7MJS, 6C08
Model resolution (Å)	2.90	3.55	3.37	4.06	3.45	3.53
FSC threshold	0.50	0.50	0.50	0.50	0.50	0.50
Model resolution range (Å)	2.48-3.72	2.84-4.50	2.90-4.77	3.39-7.00	2.92-4.36	2.90-4.56
Map sharpening *B* factor (Å^2)	-123	-114	-116	-220	-115	-115
Model composition						
Non-hydrogen atoms	7071	6609	6862	6388	6858	6813
Protein residues	879	864	864	864	864	864
Ligands	20	10	17	9	17	14
B factors (Å^2)						
Protein	19.146	39.723	71.985	84.118	66.955	38.552
Ligand(s) (ZGS)	31.153	--	44.68	73.54	55.389	27.92
Ligand(s) (LMT)	70.579	54.872	90.372	87.563	85.232	55.269
R.m.s. deviations						
Bond lengths (Å)	0.003	0.004	0.005	0.004	0.004	0.004
Bond angles (°)	0.658	0.715	0.866	0.745	0.781	0.856
Validation						
MolProbity score	1.72	1.78	1.79	1.74	1.8	1.79
Clashscore	6.89	6.46	6.69	6.08	6.7	6.32
Poor rotamers (%)	0.80	0.51	0.99	0.75	0.84	0.34
Ramachandran plot						
Favored (%)	94.96	93.11	93.46	93.81	93.34	93.22
Allowed (%)	5.04	6.81	6.54	6.19	6.54	6.66
Disallowed (%)	0	0	0	0	0.12	0.12

Supplementary Table 2

Intermolecular interactions	Merged Ligands (Ligand$_{1A, 2B, 3C}$) model	Ligand$_{1A}$ model	Ligand$_{1B}$ model
Ligand$_{1A}$ –drMfsd2a		X	
Ligand$_{1B}$-drMfsd2a			X
Ligand$_{2B}$-drMfsd2a	X		
Ligand$_{3C}$-drMfsd2a	X		
