# Supplementary material for: Lipid flipping in the omega-3 fatty-acid transporter
Source: Nat Commun. 2023 May 8;14:2571. doi: 10.1038/s41467-023-37702-7 (PMC10167227; doi:10.1038/s41467-023-37702-7)
Supplement: Supplementary file 3 — Description of Additional Supplementary Files [file 41467_2023_37702_MOESM3_ESM.pdf]

Description of Additional Supplementary Files

Supplementary Movies

Supplementary Movie 1. Interdomain contacts and rocker-switch movement between the inward and outward conformation of Mfsd2a. a-b, Morphing of the outward mouse[33] to inward drMfsd2a structure was accomplished by the Morph function in PyMol. Van der Waals interactions (grey), salt bridges (pink), and hydrogen bonds (pink) that make up the interdomain contacts are highlighted. N- and C- domains are in light blue and wheat. The $Lysolipid_{1A}$ is in teal stick and sphere representation.

Supplementary Movie 2. Transition between the outward, outward-occluded, and inward Mfsd2a structures. a-d, Side view of the structure alignment between the outward mouse[31] (grey) and outward-occluded human[32] (light cyan) Mfsd2a structures **(a, c)** and the outward-occluded human[32] (light cyan) and drMfsd2a (teal) structures **(b, d). e-h,** Extracellular view of the structure alignment between the outward mouse[31] (grey) and outward-occluded human[32] (light cyan) Mfsd2a structures **(e, g)** and the outward-occluded human[32] (light cyan) and drMfsd2a (teal) structures **(f, h). i-l,** Cytoplasmic view of the structure alignment between the outward mouse[31] (grey) and outward-occluded human[32] (light cyan) Mfsd2a structures **(i, k)** and the outward-occluded human[32] (light cyan) and drMfsd2a (teal) structures **(j, l).**

Supplementary Movie 3. Moving figures of protein conformational changes and lipid flipping, translocation, and release by drMfsd2a. a, Conformational changes during the rotation of the lipid tail from the outward-facing mouse[33] (PDB 7n98) ALA-LPC docked model to the inward-facing drMfsd2a bound with $Lysolipid_{1A}$. **b,** Conformational changes during rotation of the LPC headgroup from the $Lysolipid_{1A}$ to the $Lysolipid_{1B}$ position. **c,** The translocation and cytoplasmic release of the lipid-LPC from the $Lysolipid_{2B}$ to the $Lysolipid_{3C}$ position. **d,** The overall conformational changes from the outward-facing ALA-LPC docked to the release of $Lysolipid_{3C}$ position.
