# Supplementary material for: Lipid flipping in the omega-3 fatty-acid transporter
Source: Nat Commun. 2023 May 8;14:2571. doi: 10.1038/s41467-023-37702-7 (PMC10167227; doi:10.1038/s41467-023-37702-7)
Supplement: Supplementary file 5 — Supplementary Movie 2 [file 41467_2023_37702_MOESM5_ESM.pptx]

## Slide 1
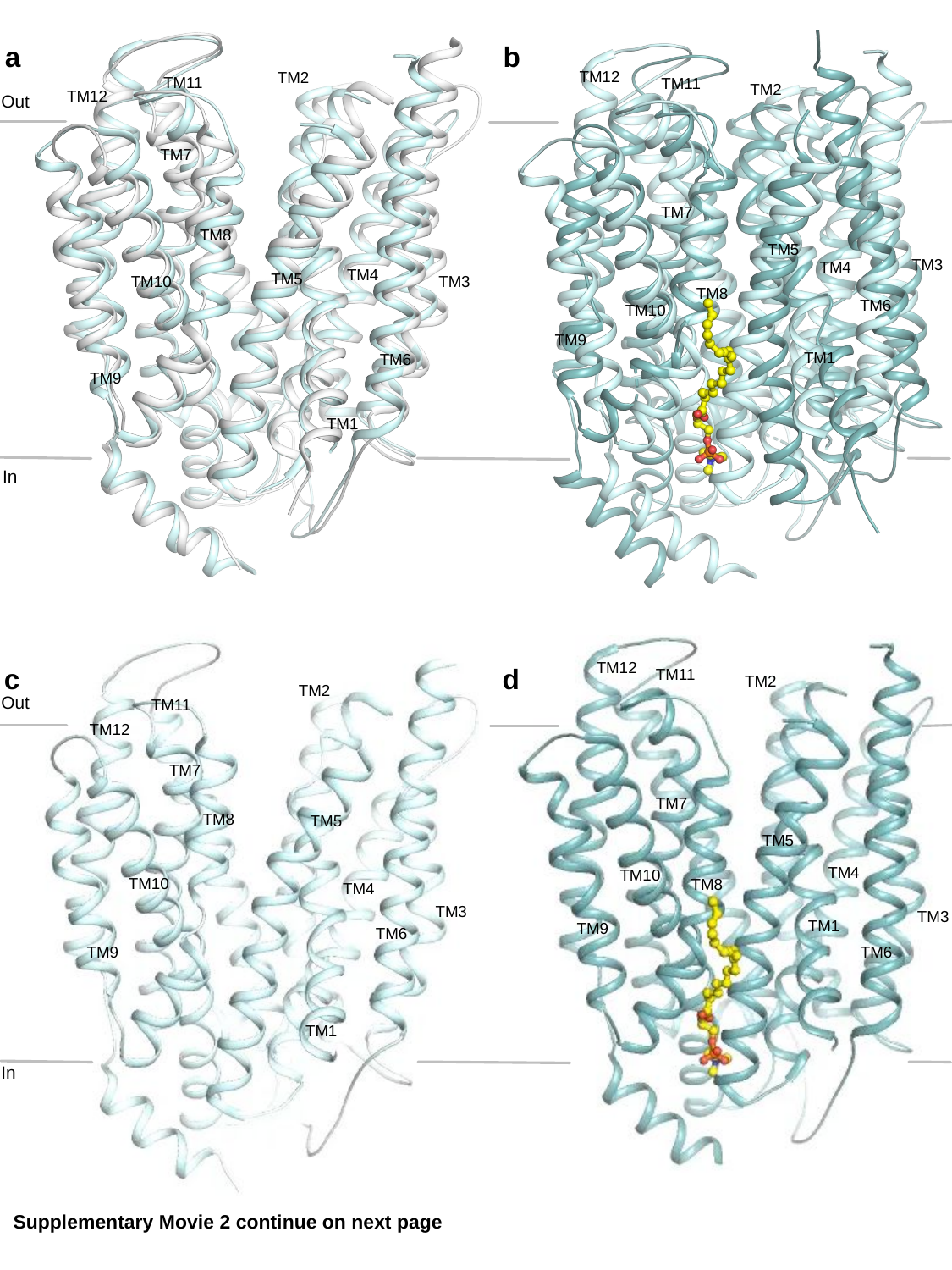

a
b
TM12
TM2
TM11
TM11
TM2
TM12
Out
TM7
TM7
TM8
TM5
TM3
TM4
TM4
TM5
TM10
TM3
TM8
TM6
TM10
TM9
TM1
TM6
TM9
TM1
In
TM12
c
d
TM11
TM2
TM2
Out
TM11
TM12
TM7
TM7
TM8
TM5
TM5
TM4
TM10
TM10
TM8
TM4
TM3
TM3
TM1
TM9
TM6
TM9
TM6
TM1
In
Supplementary Movie 2 continue on next page

## Slide 2
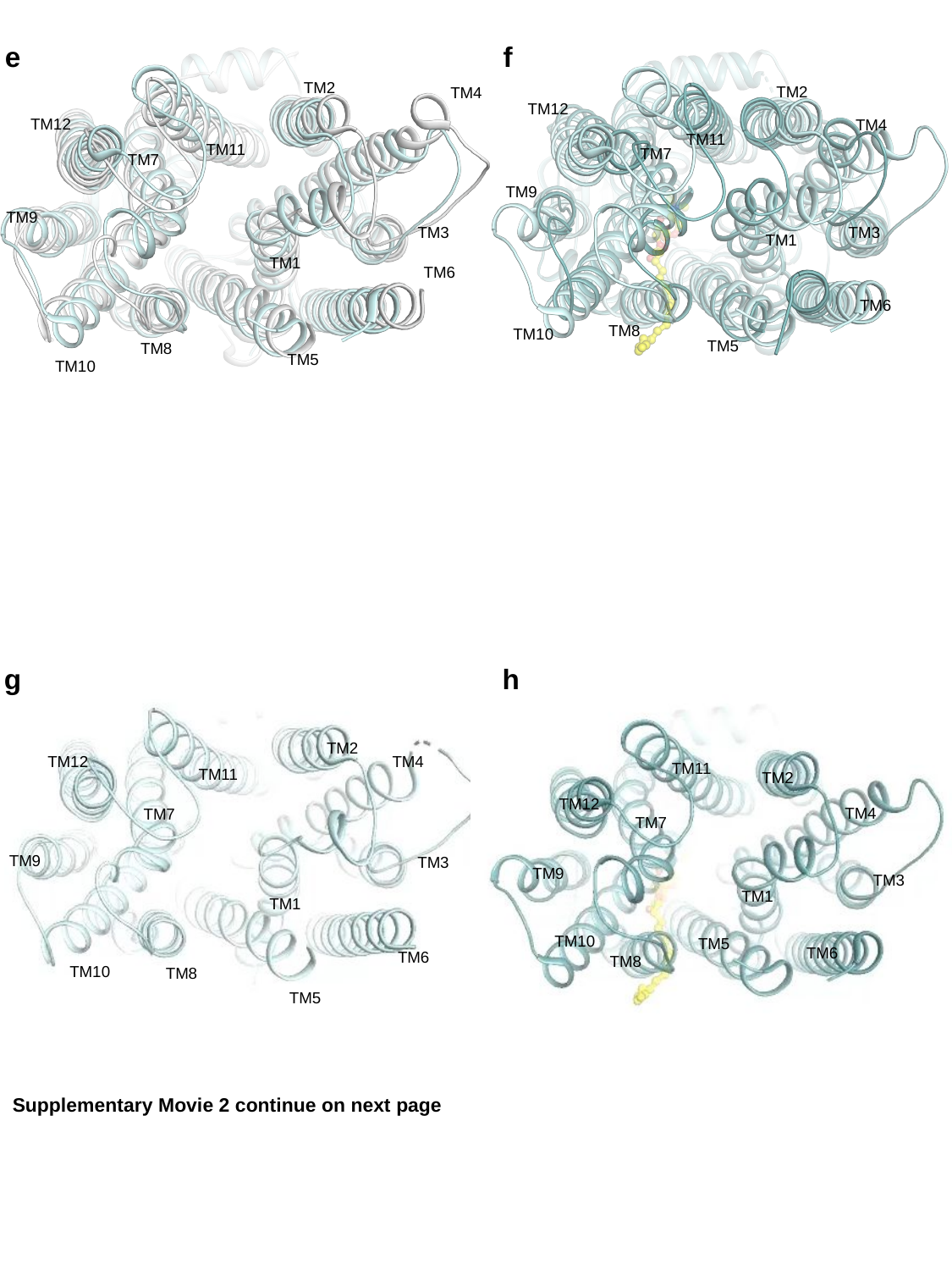

e
f
TM2
TM2
TM4
TM12
TM12
TM4
TM11
TM11
TM7
TM7
TM9
TM9
TM3
TM3
TM1
TM1
TM6
TM6
TM8
TM10
TM5
TM8
TM5
TM10
g
h
TM2
TM12
TM4
TM11
TM11
TM2
TM12
TM4
TM7
TM7
TM9
TM3
TM9
TM3
TM1
TM1
TM10
TM5
TM6
TM6
TM8
TM10
TM8
TM5
Supplementary Movie 2 continue on next page

## Slide 3
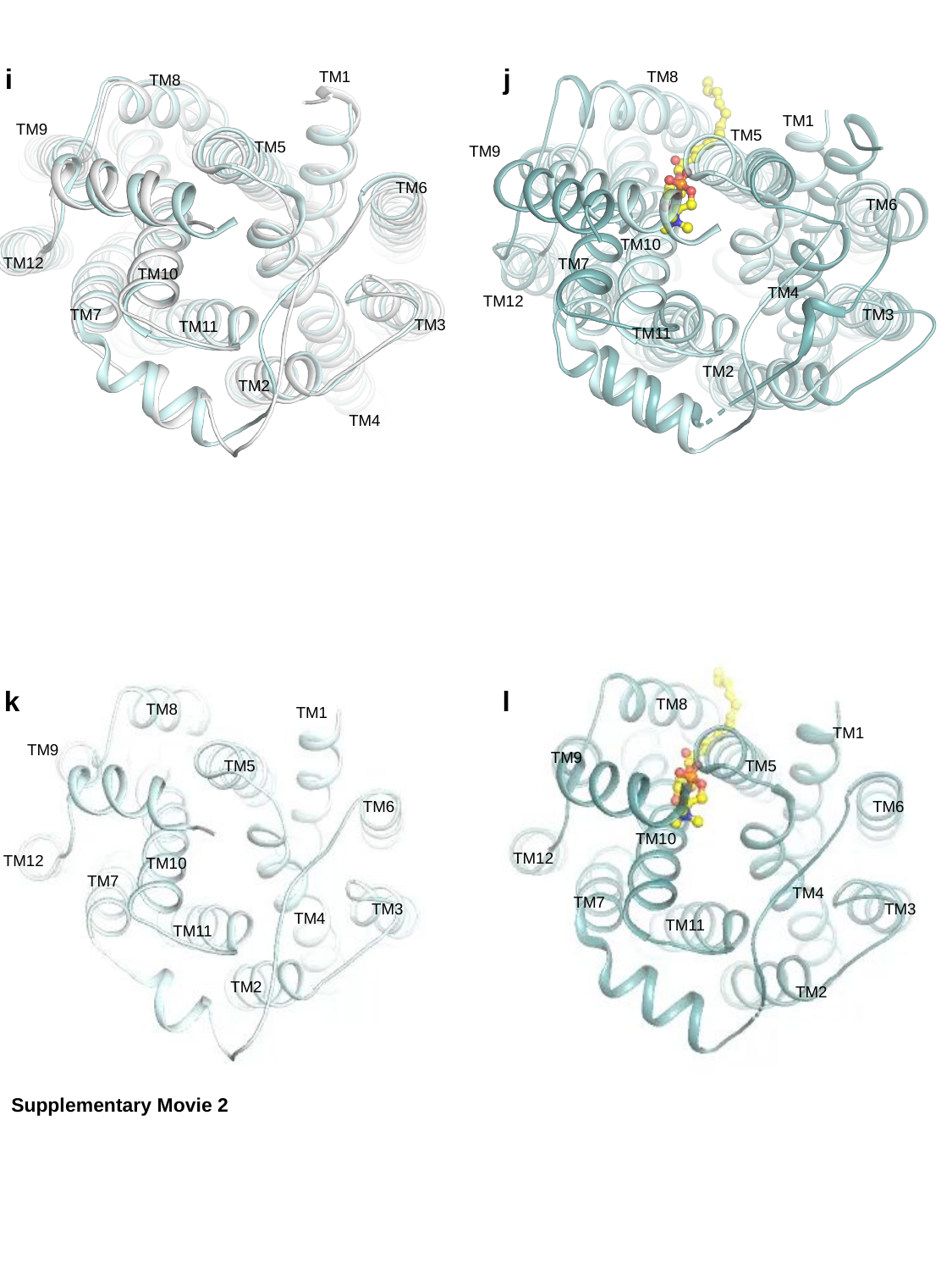

i
j
TM8
TM1
TM8
TM1
TM9
TM5
TM5
TM9
TM6
TM6
TM10
TM12
TM7
TM10
TM4
TM12
TM7
TM3
TM3
TM11
TM11
TM2
TM2
TM4
k
l
TM8
TM8
TM1
TM1
TM9
TM9
TM5
TM5
TM6
TM6
TM10
TM12
TM12
TM10
TM7
TM4
TM7
TM3
TM3
TM4
TM11
TM11
TM2
TM2
Supplementary Movie 2
