# Supplementary material for: Lipid flipping in the omega-3 fatty-acid transporter
Source: Nat Commun. 2023 May 8;14:2571. doi: 10.1038/s41467-023-37702-7 (PMC10167227; doi:10.1038/s41467-023-37702-7)
Supplement: Supplementary file 6 — Supplementary Movie 3 [file 41467_2023_37702_MOESM6_ESM.pptx]

## Slide 1
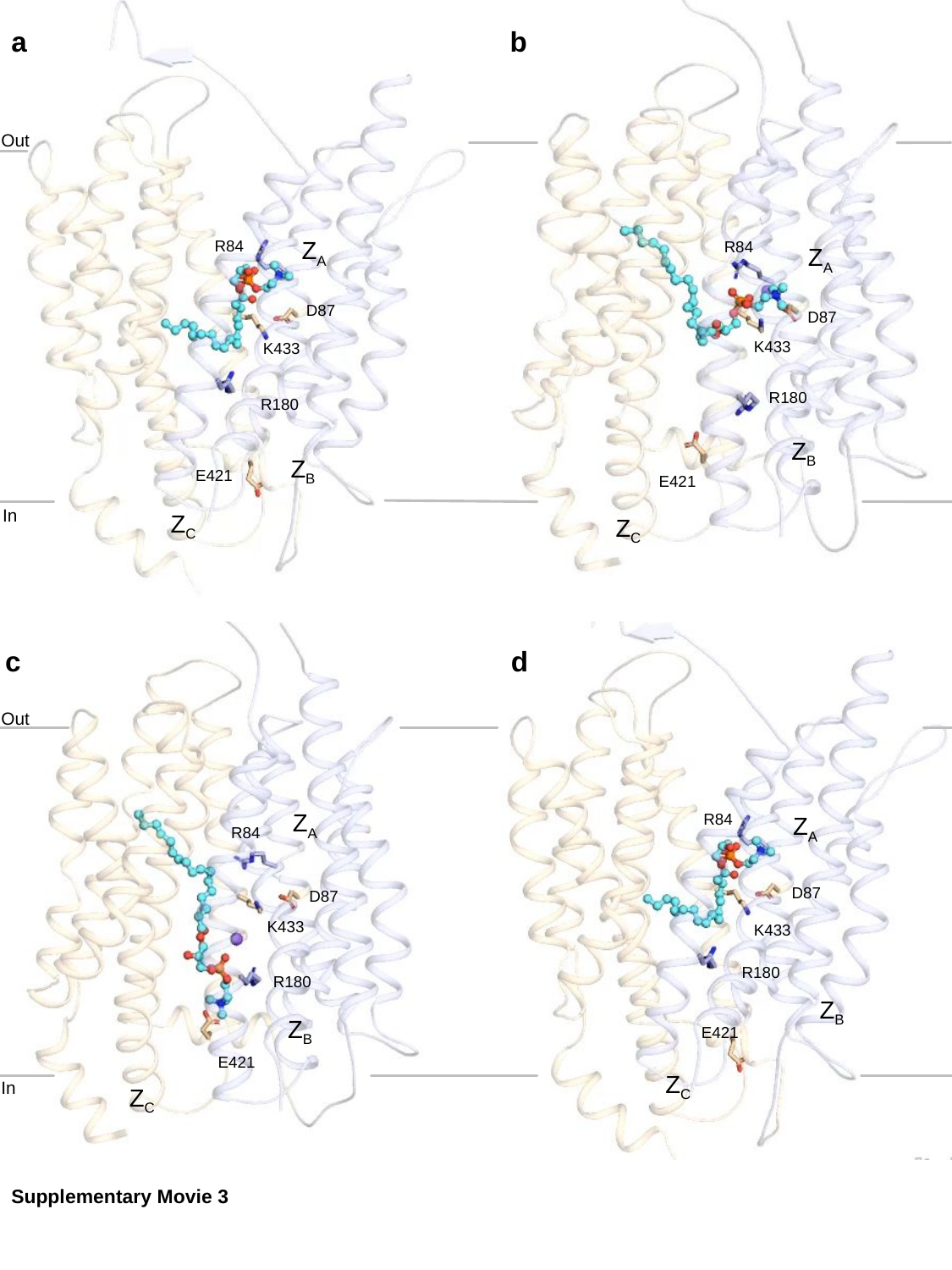

a
b
Out
ZA
R84
R84
ZA
D87
D87
K433
K433
R180
R180
ZB
ZB
E421
E421
In
ZC
ZC
c
d
Out
ZA
R84
ZA
R84
D87
D87
K433
K433
R180
R180
ZB
ZB
E421
E421
ZC
In
ZC
Supplementary Movie 3
